# Long-term psychological profile of general population following COVID-19 outbreak: symptom trajectories and evolution of psychopathological network

**DOI:** 10.1017/S2045796022000518

**Published:** 2022-09-27

**Authors:** Zheng-An Lu, Le Shi, Jian-Yu Que, Yong-Bo Zheng, Qian-Wen Wang, Wei-Jian Liu, Yue-Tong Huang, Jie Shi, Yan-Ping Bao, Lin Lu

**Affiliations:** 1 Peking University Sixth Hospital, Peking University Institute of Mental Health, NHC Key Laboratory of Mental Health (Peking University), National Clinical Research Center for Mental Disorders (Peking University Sixth Hospital), Chinese Academy of Medical Sciences Research Unit (No. 2018RU006), Peking University, Beijing, China; 2 Peking-Tsinghua Center for Life Sciences and PKU-IDG/McGovern Institute for Brain Research, Beijing, China; 3 National Institute on Drug Dependence and Beijing Key Laboratory of Drug Dependence, Peking University, Beijing, China

**Keywords:** COVID-19, general population, long term, psychological profile

## Abstract

**Aims:**

COVID-19 has long-term impacts on public mental health, while few research studies incorporate multidimensional methods to thoroughly characterise the psychological profile of general population and little detailed guidance exists for mental health management during the pandemic. This research aims to capture long-term psychological profile of general population following COVID-19 by integrating trajectory modelling approaches, latent trajectory pattern identification and network analyses.

**Methods:**

Longitudinal data were collected from a nationwide sample of 18 804 adults in 12 months after COVID-19 outbreak in China. Patient Health Questionnaire-9, Generalised Anxiety Disorder-7 and Insomnia Severity Index were used to measure depression, anxiety and insomnia, respectively. The unconditional and conditional latent growth curve models were fitted to investigate trajectories and long-term predictors for psychological symptoms. We employed latent growth mixture model to identify the major psychological symptom trajectory patterns, and ran sparse Gaussian graphical models with graphical lasso to explore the evolution of psychopathological network.

**Results:**

At 12 months after COVID-19 outbreak, psychological symptoms generally alleviated, and five psychological symptom trajectories with different demographics were identified: normal stable (63.4%), mild stable (15.3%), mild-increase to decrease (11.7%), mild-decrease to increase (4.0%) and moderate/severe stable (5.5%). The finding indicated that there were still about 5% individuals showing consistently severe distress and approximately 16% following fluctuating psychological trajectories, who should be continuously monitored. For individuals with persistently severe trajectories and those with fluctuating trajectories, central or bridge symptoms in the network were mainly ‘motor abnormality’ and ‘sad mood’, respectively. Compared with initial peak and late COVID-19 phase, aftermath of initial peak might be a psychologically vulnerable period with highest network connectivity. The central and bridge symptoms for aftermath of initial peak (‘appetite change’ and ‘trouble of relaxing’) were totally different from those at other pandemic phases (‘sad mood’).

**Conclusions:**

This research identified the overall growing trend, long-term predictors, trajectory classes and evolutionary pattern of psychopathological network of psychological symptoms in 12 months after COVID-19 outbreak. It provides a multidimensional long-term psychological profile of the general population after COVID-19 outbreak, and accentuates the essentiality of continuous psychological monitoring, as well as population- and time-specific psychological management after COVID-19. We believe our findings can offer reference for long-term psychological management after pandemics.

## Introduction

COVID-19, which caused over 500 million infections and 6 million deaths globally, has put the whole society under tremendous mental health strain (World Health Organization, WHO Coronavirus Dashboard, accessed on 5 August 2022). The continuous emergence of sporadic cases, frequent local resurgences, long-term pandemic control measures and socio-economic repercussions are all psychological stressors posing enduring threats to public mental health (Brooks *et al*., 2020; Hiremath *et al*., 2020; Zhou *et al*., 2020; Wang *et al*., 2021). Preliminary research conducted during COVID-19 outbreak indicated potential mental impacts of COVID-19, complying with findings from previous pandemics (Maunder *et al*., 2006; Lee *et al*., 2007; Holmes *et al*., 2020; Shi *et al*., 2020, 2021*a*, 2021*b*; Zheng *et al*., 2021). However, since psychological stressors may exert distinct influences on people across different pandemic phases, longitudinal studies are required to capture complete psychological trajectories after COVID-19 so as to provide reference for long-term psychological management (Fancourt *et al*., 2021).

Existing longitudinal studies based on large samples have unveiled different psychological symptom growth curves and trajectory patterns over the course of COVID-19, most of which indicated increase in distress at initial peak and symptom plateauing or alleviation during lockdown (Iob *et al*., 2020; Planchuelo-Gómez *et al*., 2020; Salfi *et al*., 2020; Fancourt *et al*., 2021; Prati and Mancini, 2021; Ripoll *et al*., 2021; Saunders *et al*., 2021*a*, 2021*b*). However, all these studies employed traditional single-dimensional approaches that could not offer detailed information for psychological management at different pandemic phases. Another important question to answer is whether some populations suffer more adverse psychological impacts from COVID-19. Existing studies have identified some demographic predictors (e.g. young age, females, low socioeconomic status, poor physical or mental health status) and epidemic-related predictors (e.g. being infected, working as essential workers, quarantine, trauma history, exposure risk) for long-term psychological symptoms after pandemics (Maunder *et al*., 2006; Lee *et al*., 2007; Liu *et al*., 2012; Iob *et al*., 2020; Saunders *et al*., 2021*b*; Shi *et al*., 2021*a*). However, these studies failed to quantify effect sizes of predictors at different pandemic phases, which limited their potentials in guiding time-specific interventions. Studies based on large representative samples, with longer observational periods, and incorporating multidimensional psychological modelling approaches are in urgent demand to offer reference for population- and time-specific mental health management.

Person-centred trajectory modelling approaches (i.e. latent growth curve models, LGCMs) that consider individual differences when estimating trajectories can reveal a more reliable developmental trend for psychological symptoms (Felt *et al*., 2017). Conditional LGCMs may quantify not only the effects of predictors on symptom baseline level, but also on symptom changing slopes (Lu *et al*., 2022). Further, some advanced models developed in recent years may offer in-depth information by incorporating novel perspectives of latent pattern identification and graph theory. Latent growth mixture model (LGMM), a person-centred clustering method, can extract the major psychological symptom trajectory patterns after COVID-19 from a heterogeneous population (Hannigan *et al*., 2018). Characterising the demographics of these trajectory patterns can provide information for population-specific mental health management. In addition, the ‘psychopathological network’ analysis, which assumes mental disorders as networks based on causal interactions among individual symptoms, can provide insights into psychopathological structures and potential treatment target symptoms across different pandemic phases and populations in a trans-diagnostic perspective (Bringmann *et al*., 2022). Specifically, the global network connectivity can reflect overall susceptibility to mental disorders of a population, and previous studies reveal that patients with densely connected network have worse prognosis (Borsboom and Cramer, 2013; O'Driscoll *et al*., 2021). The central symptom and the bridge symptom can be highly prospective treatment targets for emergence and comorbidity of mental disorders (Opsahl *et al*., 2010; Borsboom and Cramer, 2013; Hallquist *et al*., 2021). Adopting an integrative approach by combining these multidimensional methods can greatly enrich our understanding of long-term psychopathology of the general population following pandemics so as to provide reference for psychological management.

The aim of the current study is to capture the multidimensional long-term psychological profile of the general population after COVID-19 outbreak by integrating symptom trajectory modelling, latent pattern identification and psychopathological network analyses based on repeatedly collected data from a nationwide sample of 18 804 adults in China, to provide population- and time-specific reference for mental health management after pandemics.

## Methods

### Procedures and participants

We conducted a longitudinal observational study via Joybuy, a large digital commercial clustering website with 0.44 billion active users from all 34 provincial regions in China, as detailed elsewhere (Shi *et al*., 2021*a*; Wang *et al*., 2021).

We fielded three surveys after COVID-19 outbreak. Survey 1 was fielded during initial COVID-19 peak and lockdown period (28 February 2020 to 11 March 2020). Survey 2 was fielded in the aftermath of initial COVID-19 peak (8 July 2020 to 8 August 2020), when main wave had been controlled and lockdown had ended, but sporadic cases and local resurgences were common, and pandemic control measures were changing frequently. Survey 3 was fielded during late COVID-19 phase (29 January 2021 to 26 April 2021), when daily new confirmed cases were few and most pandemic control measures were removed. During survey 1, we adopted an untargeted approach to recruit participants, by posting a survey link on Joybuy and allowing participants to voluntarily click on it until the sample represented all 34 provincial regions in China, as detailed elsewhere (Shi *et al*., 2020). During surveys 2 and 3, we adopted both targeted and untargeted approaches to recruit participants. In the targeted approach, we sent survey links to all participants who had completed at least one previous survey via the message platform of Joybuy. In the untargeted approach, we followed the same recruiting procedure as survey 1.

**Fig. 1.**
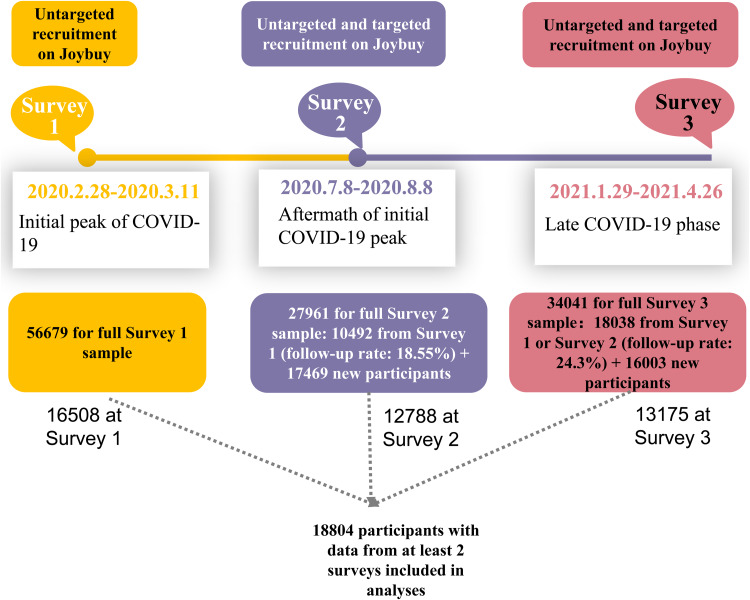
Flow graph for participants recruitment at three surveys.

All participants were registered members of Joybuy. During survey 1, 56 932 of 71 227 participants who clicked on survey links provided informed consent and completed the survey, with an effective response rate of 79.9%. After quality control, 56 679 adults with valid age information comprised the final survey 1 sample, as detailed elsewhere (Shi *et al*., 2020). After excluding all participants under 18 or with invalid age information, the final survey 2 and 3 samples comprised of 27 961 and 34 041 adults, respectively. The final longitudinal sample comprised of 18 804 adults with valid data from at least two of the three surveys. Of the 18 804 participants, data were available for 16 508 in survey 1, 12 788 in survey 2 and 13 175 in survey 3. Detailed information is presented in Fig. 1 and in previous research (Lu *et al*., 2022).

### Measures

We designed questionnaires to collect information on demographics, epidemic-related information, quarantine conditions, as well as to measure symptoms of depression, anxiety and insomnia in three surveys. The detailed contents of questionnaires were provided in previous research (Shi *et al*., 2020, 2021*a*). Chinese versions of Patient Health Questionnaire-9 (PHQ-9), Generalised Anxiety Disorder-7 (GAD-7) and Insomnia Severity Index (ISI) were used to measure depression, anxiety and insomnia, respectively (Kroenke *et al*., 2001; Löwe *et al*., 2008; Gagnon *et al*., 2013).

### Variables

To explore long-term predictors for depression, anxiety and insomnia, we considered the following three categories of variables: (1) demographic variables: gender, age, living area, educational level, marital status, monthly family income, history of chronic diseases, history of psychiatric disorders, family history of psychiatric disorders, history of sleep disturbance, history of smoking and history of alcohol abuse; (2) variables related to COVID-19 infection: COVID-19 patients, family members of COVID-19 patients, close contacts of COVID-19 patients, engagement in COVID-19-related work, family members of workers directly engaging in COVID-19 control and occupational exposure risk to COVID-19; (3) variables related to post-pandemic repercussions: quarantine, living in places severely affected by initial peak, local resurgences, increases in workloads, unemployment due to COVID-19, seeking psychological consultation, wearing face masks and reducing social gatherings. Variables were dichotomised based on participants' responses to questionnaire questions, and variable constructing approaches were described elsewhere (Shi *et al*., 2021*a*).

### Statistical analyses

Descriptive statistics were used to present the demographic characteristics of the longitudinal sample. To explore trajectories, predictors, symptom trajectory patterns and evolution of psychopathological networks of depression, anxiety and insomnia, we conducted the following four analyses.

In the first step, to explore developmental trajectories of depression, anxiety and insomnia, we conducted LGCM analyses. Given the high correlation among three symptoms, we conducted a multi-process LGCM analysis, in which depression, anxiety and insomnia scores were simultaneously entered into one model and paths between symptoms were incorporated, so that the general symptom growing trend could be estimated (Hannigan *et al*., 2018; Fancourt *et al*., 2021). Next, we fitted three independent LGCMs, in which outcome variables were depression, anxiety and insomnia scores respectively, so that trajectories for the three single symptoms could be estimated. All variables considered were adjusted for in each model. For each model, we tested two types of growth factors: intercept-only and linear slope (Hannigan *et al*., 2018). For the models with linear-slope growth factor, we further tested fixed (coded as 0, 5 and 12 in three surveys) and free slope factor loadings. We determined the optimal models based on comparative fit index (CFI), chi-square, standardised root-mean-square residual (SRMR) and the root mean square error of approximation (RMSEA) statistics (Hu and Bentler, 1999). Smaller values for chi-square, RMSEA, SRMR and larger values for CFI indicate a better fit. Following established recommendations, good fit was considered when CFI ⩾0.95, RMSEA ⩽0.05 and SRMR ⩽0.05 (Hannigan *et al*., 2018; Pavlov *et al*., 2021). Mean for intercept reflects the average psychological symptom score at survey 1, and mean for slope reflects the average change in psychological symptom score from survey 1 to survey 3 (Hu and Bentler, 1999).

In the second step, to identify predictors of depression, anxiety and insomnia trajectories, we fitted three independent conditional linear slope LGCMs, in which outcome variables were depression scores, anxiety scores and insomnia scores, respectively. All variables considered were added into the model as independent variables, so that the growth factors (i.e. intercepts and slopes) could be regressed on these variables. In the conditional LGCM, the mean effect (*B* value) for intercept reflects the predicting effect of a variable on psychological symptom scores in survey 1, while the mean effect (*B* value) for slope reflects the predicting effect of a variable on longitudinal changes in scores from survey 1 to survey 3.

In the third step, to explore the psychological symptom trajectory patterns throughout COVID-19, we conducted a LGMM analysis. Considering high correlation among three symptoms, we employed a joint trajectory approach, which was previously used to identify co-developing trajectory patterns for several highly correlated symptoms (Hannigan *et al*., 2018). Scores for depression, anxiety and insomnia were all entered into one single LGMM as outcome variables, so that co-developing patterns for the three symptoms could be unveiled. We gradually added number of latent classes from 2 to 11, and determined the optimal number of latent classes mainly based on Vuong–Lo–Mendell–Rubin likelihood ratio test (VLMR-LRT) (Lo *et al*., 2001), with due consideration of parsimony, interpretability, Akaike information criterion (AIC) (Akaike, 1981), Bayesian information criterion (BIC) (Schwartz, 1978), adjusted BIC (aBIC) (Sclove, 1987) and entropy (Lo *et al*., 2001). VLMR-LRT was considered as an acceptable method to determine optimal number of classes (Lo *et al*., 2001). A *p* value lower than 0.05 in VLMR-LRT suggests a better fit of model with *k* trajectory classes compared with model with *k* − 1 trajectory classes (Lo *et al*., 2001). Lower BIC, aBIC and AIC values indicate a better fit (Schwartz, 1978; Akaike, 1981; Sclove, 1987). Entropy characterises quality of classification on a 0–1 scale (Celeux and Soromenho, 1996). Entropy value closer to 1 indicates clear delineation of classes (Celeux and Soromenho, 1996) and 0.80 is often used as an acceptable level (Greenwood *et al*., 2019). After determining optimal number of latent trajectory classes, all individuals in the longitudinal sample acquired their class membership based on posterior probability. We conducted multinomial logistic regression of latent trajectory class membership on predictors using the three-step procedure (R3STEP) in Mplus software (Bakk *et al*., 2013). In the three steps above, all missing data were handled with full information maximum likelihood estimation, which supposed that missingness was at random (Cham *et al*., 2017). The significance level was set to two-sided *p* < 0.05. All statistical analyses were performed in SPSS 22 software (SPSS, Chicago, IL, USA), Mplus 8.3 and R version 4.0.3.

In the fourth step, to estimate the psychopathological network for depression, anxiety and insomnia in three surveys, we ran three sparse Gaussian graphical models with graphical lasso on eight items from Patient Health Questionnaire-9 (excluding sleep disturbances, overlapping with other scales), seven items from Generalised Anxiety Disorder-7 and three items form Insomnia Severity Index (excluding four non-symptom items) (Friedman *et al*., 2008). The tuning parameters were determined by extended BIC (van Borkulo *et al*., 2014). In the psychopathological network, scores of items were considered as ‘nodes’, and pair-wise correlations between items were considered as ‘edges’ (Borsboom and Cramer, 2013). Global connectivity of network can be characterised by ‘global strength’ defined as sum of edge weights in the whole network. Higher global connectivity indicates greater susceptibility to mental disorders. The network structures can be characterised by network centrality and bridge centrality. Network centrality indices include strength, closeness, betweenness and expected influence, which can reflect the place of each node in the network. Expected influence is defined as sum of edge weights directly connected to a node, and is regarded as optimal indicator for centrality in a network with both positive and negative edges (Opsahl *et al*., 2010). Bridge centrality indices include bridge strength, bridge closeness, bridge betweenness and bridge expected influence, which can reflect the ability of each node in connecting different disorders. Bridge expected influence is defined as sum of edge weights connecting a node to all nodes from other disorders, and is commonly considered as an optimal indicator for the bridge centrality (Opsahl *et al*., 2010). The central symptom, defined as the symptom with greatest centrality, can be a potential treatment target for mental disorders (Opsahl *et al*., 2010; Borsboom and Cramer, 2013). The bridge symptom, defined as the symptom with greatest bridge centrality, can be a highly prospective treatment target for comorbidity of mental disorders. In this research, central symptom and bridge symptom were determined by values of expected influence and bridge expected influence, respectively (Opsahl *et al*., 2010; Borsboom *and* Cramer, 2013). Networks were also estimated in identified psychological symptom trajectory classes with the same procedures and parameters described above. The R package ‘bootnet’ was utilised to estimate the psychopathological network and evaluate the network stability (Epskamp *et al*., 2018). The R package ‘NetworkComparisonTest’ was used to calculate and compare the global connectivity of networks in three pandemic phases. The R packages ‘qgraph’ and ‘networktools’ were utilised to calculate the network centrality indices and bridge centrality indices, respectively. The network analyses were performed with R version 4.0.3.

## Results

### Demographic characteristics of the longitudinal sample

The 18 804 participants had a mean (s.d.) age of 36.6 (8.2) years, among whom 8558 (45.4%) were males, 17 599 (93.6%) lived in urban areas, 15 489 (82.4%) had a college school or higher educational level, 14 783 (78.6%) were married and 4186 (22.3%) had a monthly family income lower than 5000 yuan, as is presented in online Supplementary Table S1.

### Trajectories and predictors of psychological symptoms after COVID-19 outbreak

**Fig. 2.**
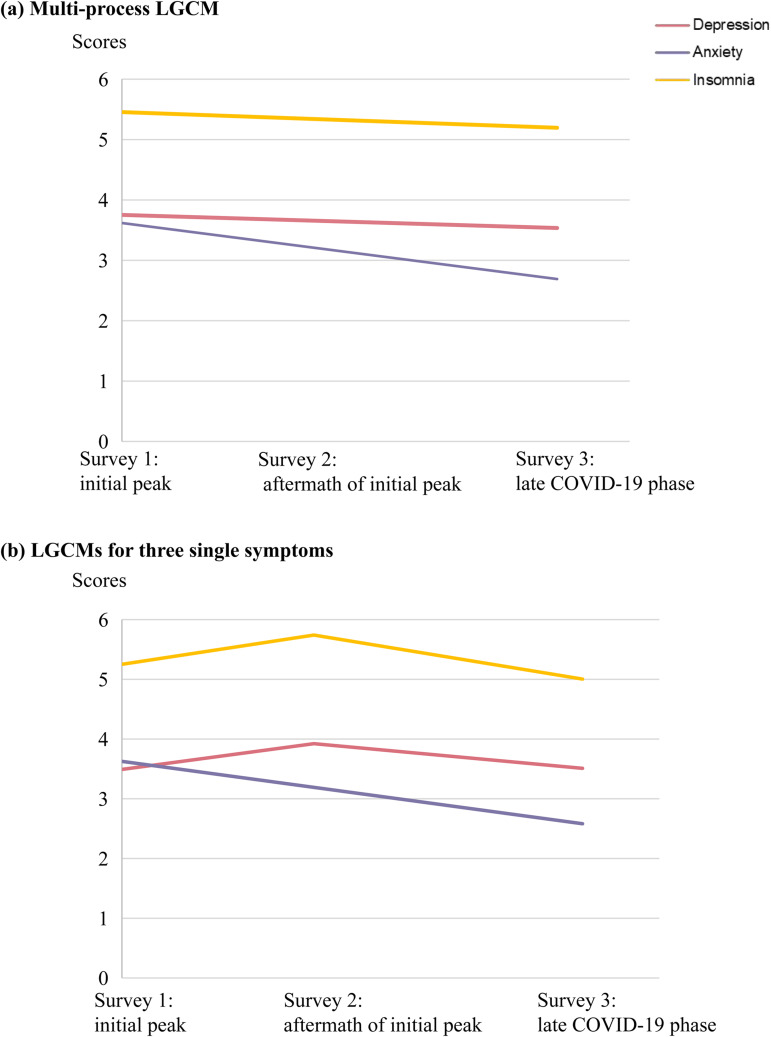
Predicted trajectories of depression, anxiety and insomnia from the best fitting: (*a*) multi-process LGCM and (*b*) LGCMs for three single symptoms.

Modelling fitting statistics for LGCMs with different growth factors are presented in online Supplementary Table S2. The multi-process LGCM revealed that psychological symptoms generally decreased [depression, estimated mean (s.e.) for slope −0.09 (0.02), *p* < 0.001; anxiety, −0.39 (0.02), *p* < 0.001; insomnia, −0.11 (0.02), *p* < 0.001; Fig. 2*a*]. Specifically, the best-fitting model for depression indicated a mild worsening trend, in which the mean (s.e.) for slope was 0.43 (0.05, *p* < 0.001). However, the best-fitting models for anxiety and insomnia symptoms suggested slight alleviating trends. Mean (s.e.) for slope was −0.44 (0.02, *p* < 0.001) for anxiety and −0.49 (0.03, *p* < 0.001) for insomnia (Fig. 2*b*).

In addition, we identified demographic factors, infection-related factors and post-COVID-19 repercussions as long-term predictors for intercepts or slopes of psychological trajectories after COVID-19. Significant predictors are illustrated in online Supplementary Fig. S1 and Table 1.

**Table 1.** Predictors for intercepts and slopes of depression, anxiety and insomnia scores from the conditional LGCMs

Predictors	Depression: intercept		Depression: slope		Anxiety: intercept		Anxiety: slope		Insomnia: intercept		Insomnia: slope	
	*B* (s.e.)	*p*	*B* (s.e.)	*p*	*B* (s.e.)	*p*	*B* (s.e.)	*p*	*B* (s.e.)	*p*	*B* (s.e.)	*p*
Demographic predictors												
Gender: male (*v*. female)	0.58 (0.08)	<0.001	0.12 (0.11)	0.30	0.05 (0.08)	0.54	0.18 (0.06)	0.002	0.24 (0.07)	0.001	−0.13 (0.07)	0.06
Age group: 18–39 (*v*. ⩾40)	0.60 (0.08)	<0.001	0.04 (0.10)	0.71	0.28 (0.08)	<0.001	0.09 (0.06)	0.11	−0.08 (0.07)	0.29	−0.04 (0.07)	0.54
Marital status: married (*v*. unmarried)	−0.31 (0.09)	0.001	−0.30 (0.13)	0.02	0.06 (0.09)	0.50	−0.12 (0.07)	0.11	−0.81 (0.09)	<0.001	−0.09 (0.08)	0.24
Monthly family income (CNY): 0–4999 (*v*. >5000)	0.35 (0.09)	<0.001	−0.10 (0.13)	0.43	0.30 (0.09)	0.001	−0.06 (0.07)	0.40	0.01 (0.08)	0.94	0.02 (0.08)	0.82
History of psychiatric disorders: yes (*v*. no/unknown)	2.29 (0.63)	<0.001	1.35 (0.85)	0.11	1.57 (0.79)	0.05	−0.03 (0.61)	0.96	2.10 (0.50)	<0.001	−0.64 (0.46)	0.16
History of alcohol abuse: yes (*v*. no/unknown)	0.62 (0.14)	<0.001	−0.41 (0.21)	0.05	0.41 (0.13)	0.002	−0.03 (0.10)	0.77	0.66 (0.13)	<0.001	0.14 (0.12)	0.26
Predictors associated with COVID-19 infection												
COVID-19 patients: yes (*v*. no)	1.32 (0.53)	0.01	0.04 (0.60)	0.95	0.04 (0.52)	0.94	0.36 (0.42)	0.39	0.26 (0.42)	0.53	0.89 (0.37)	0.02
Family members of COVID-19 patients: yes (*v*. no)	1.29 (0.28)	<0.001	−0.03 (0.37)	0.93	0.90 (0.27)	0.001	−0.03 (0.21)	0.89	1.33 (0.25)	<0.001	0.07 (0.21)	0.72
Occupational exposure risk to COVID-19: yes (*v*. no)	1.60 (0.11)	<0.001	−0.19 (0.15)	0.18	0.81 (0.11)	<0.001	−0.03 (0.08)	0.72	0.41 (0.09)	<0.001	0.03 (0.09)	0.71
Engaging in COVID-19-related work: yes (*v*. no)	0.38 (0.09)	<0.001	0.05 (0.12)	0.70	0.19 (0.09)	0.03	−0.09 (0.07)	0.17	0.08 (0.08)	0.32	−0.01 (0.08)	0.94
Predictors associated with post-pandemic repercussions												
Quarantine: yes (*v*. no)	0.73 (0.08)	<0.001	0.18 (0.11)	0.09	0.50 (0.08)	<0.001	−0.05 (0.06)	0.45	0.50 (0.07)	<0.001	−0.21 (0.07)	0.002
Living in places severely affected by initial break: yes (*v*. no)	0.73 (0.20)	<0.001	−0.17 (0.26)	0.51	1.16 (0.19)	<0.001	−0.75 (0.15)	<0.001	0.25 (0.16)	0.12	−0.12 (0.15)	0.44
Living in places with COVID-19 resurgences: yes (*v*. no)	0.54 (0.08)	<0.001	0.33 (0.11)	0.004	0.37 (0.08)	<0.001	0.10 (0.07)	0.13	0.81 (0.08)	<0.001	−0.10 (0.07)	0.16
Increases in workload: yes (*v*. no)	0.84 (0.07)	<0.001	0.38 (0.10)	<0.001	0.85 (0.07)	<0.001	0.03 (0.06)	0.67	1.31 (0.07)	<0.001	−0.22 (0.06)	0.001
Unemployment due to COVID-19: yes (*v*. no)	1.52 (0.13)	<0.001	0.39 (0.16)	0.02	1.09 (0.12)	<0.001	0.02 (0.10)	0.82	1.04 (0.11)	<0.001	−0.26 (0.10)	0.01
Wearing facemasks voluntarily: yes (*v*. no)	−1.87 (0.37)	<0.001	−1.49 (0.51)	0.003	−0.79 (0.31)	0.01	−0.71 (0.29)	0.01	−1.30 (0.30)	<0.001	0.72 (0.35)	0.04
Reducing social gatherings: yes (*v*. no)	−0.53 (0.15)	<0.001	−0.48 (0.23)	0.04	−0.09 (0.14)	0.52	−0.25 (0.11)	0.02	−0.34 (0.13)	0.008	0.22 (0.15)	0.15

### Psychological symptom trajectory patterns among the general population after COVID-19 outbreak

Model fitting indicators for LGMMs with 2 to 11 latent classes are presented in online Supplementary Table S3. We selected 5-class LGMM as the optimal model mainly based on VLMR-LRT, with due consideration of parsimony, interpretability, entropy, AIC, BIC and aBIC.

**Fig. 3.**
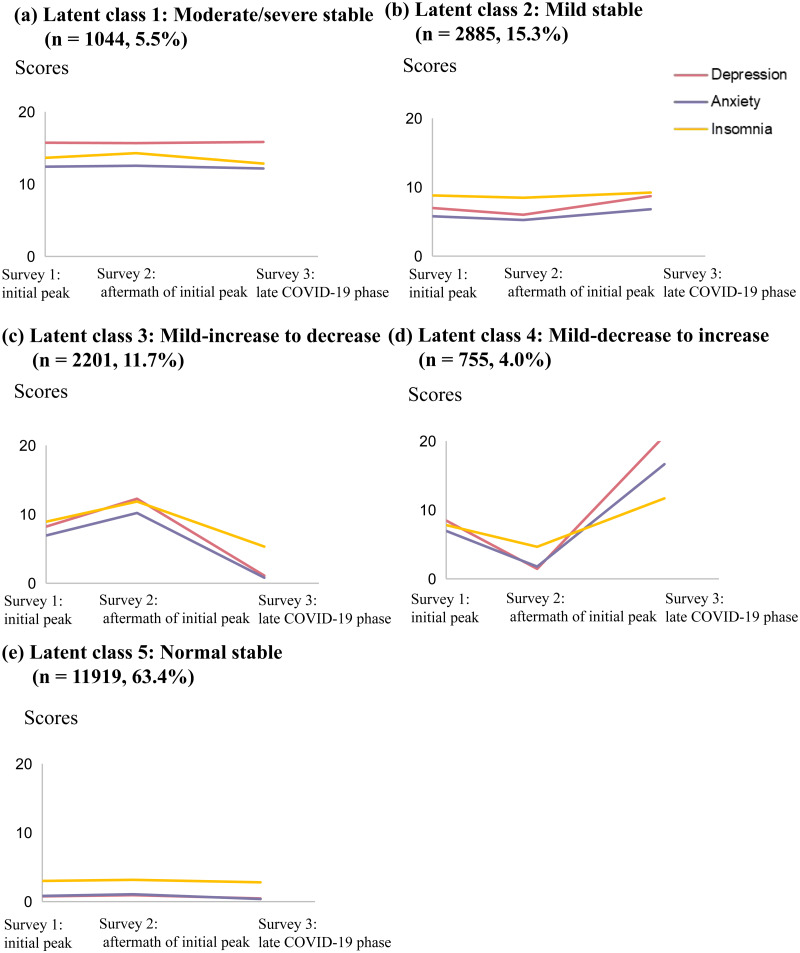
Predicted trajectories of depression, anxiety and insomnia across latent symptom trajectory classes from the best fitting 5-class LGMM. (*a*) Latent class 1: moderate/severe stable (*n* = 1044, 5.5%); (*b*) latent class 2: mild stable (*n* = 2885, 15.3%); (*c*) latent class 3: mild-increase to decrease (*n* = 2201, 11.7%); (*d*) latent class 4: mild-decrease to increase (*n* = 755, 4.0%) and (*e*) latent class 5: normal stable (*n* = 11 919, 63.4%).

We illustrated the five symptom trajectory classes from the optimal 5-class model in Fig. 3. The first class showed persistent moderate-to-severe symptoms throughout COVID-19 and accounted for 5.5% of the total sample, which was labelled as ‘moderate/severe stable’ class. In addition, there were three latent classes that demonstrated mild symptom level during initial peak, but followed distinct trajectories afterwards: the first class followed relatively stable trajectory and accounted for 15.3% of the total sample, which was labelled as ‘mild stable’ class; the second class experienced a dramatic deterioration in all three symptoms in the aftermath of initial COVID-19 peak, but came back to normal in the late COVID-19 phase, which was labelled as ‘mild-increase to decrease’ class and accounted for 11.7% of the total sample; the third class recovered to the normal level in the aftermath of initial peak, but experienced a substantial surge afterwards and ended with moderate-to-severe symptoms during late COVID-19 phase, which was labelled as ‘mild-decrease to increase’ class and accounted for 4.0% of the total sample. The last class had normal and stable psychological status throughout COVID-19 and accounted for the largest proportion (63.4%), which was labelled as ‘normal stable’ class (Fig. 3; online Supplementary Table S4).

Table 2 and online Supplementary Table S5 demonstrated the demographic characteristics of the five latent symptom trajectory classes. ‘Moderate/severe stable’ class featured highest proportion of males, youngsters, the poorly educated, the unmarried, the impoverished, individuals with history of chronic diseases or psychiatric disorders, as well as individuals severely affected by COVID-19 (i.e. quarantine, living in places severely affected by COVID-19 and COVID-19-related stressful life events). ‘Normal stable’ class distinguished itself from other four classes with its highest proportion of females, the mid-aged or elders and individuals with higher incomes. Furthermore, the two mild classes with fluctuating trajectories were more likely to report history of chronic disease or psychiatric disorders, quarantine experiences and poor education compared with ‘mild stable’ class.

**Table 2.** Multinomial logistic regression of psychological symptom trajectory class membership on predictors using a three-step approach^a^

Factors	Odds ratio [95% CI] for moderate/severe stable *v*. normal stable	*p*	Odds ratio [95% CI] for mild stable *v*. normal stable	*p*	Odds ratio [95% CI] for mild-increase to decrease *v*. normal stable	*p*	Odds ratio [95% CI] for mild-decrease to increase *v*. normal stable	*p*
Gender: male (*v*. female)	1.88 [1.61–2.15]	<0.001	1.28 [1.16–1.40]	<0.001	1.41 [1.27–1.56]	<0.001	1.20 [1.00–1.41]	0.05
Age group: 18–39 (*v*. ⩾40)	1.76 [1.45–2.06]	<0.001	1.37 [1.22–1.51]	<0.001	1.29 [1.14–1.45]	<0.001	1.35 [1.08–1.62]	0.01
Living area: urban (*v*. rural)	0.92 [0.69–1.16]	0.52	1.08 [0.87–1.29]	0.47	0.83 [0.67–1.00]	0.05	0.85 [0.57–1.12]	0.27
Educational level: college school or higher (*v*. lower than college school)	0.72 [0.59–0.85]	<0.001	1.24 [1.07–1.41]	0.005	1.10 [0.94–1.26]	0.21	0.99 [0.77–1.22]	0.95
Marital status: married (*v*. unmarried)	0.70 [0.59–0.81]	<0.001	0.81 [0.72–0.90]	<0.001	0.83 [0.73–0.94]	0.002	1.11 [0.86–1.36]	0.39
Monthly family income (CNY): 0–4999 (*v*. ⩾5000)	1.41 [1.17–1.64]	0.001	1.31 [1.16–1.45]	<0.001	1.11 [0.97–1.26]	0.13	1.18 [0.94–1.43]	0.14
History of chronic diseases: yes (*v*. no/unknown)	1.42 [1.02–1.82]	0.04	1.21 [0.99–1.44]	0.07	1.22 [0.96–1.49]	0.10	1.32 [0.87–1.77]	0.16
History of psychiatric disorders: yes (*v*. no/unknown)	11.32 [4.26–18.38]	0.004	5.58 [1.98–9.17]	0.01	7.19 [2.48–11.90]	0.01	7.68 [1.03–14.32]	0.05
Living in places severely affected by COVID-19: yes (*v*. no)^b^	2.06 [1.77–2.35]	<0.001	1.55 [1.40–1.70]	<0.001	1.27 [1.13–1.42]	<0.001	1.09 [0.88–1.29]	0.41
Quarantine: yes (*v*. no)	2.22 [1.91–2.53]	<0.001	1.26 [1.14–1.39]	<0.001	1.55 [1.38–1.71]	<0.001	1.79 [1.48–2.10]	<0.001
COVID-19-related stressful life events: yes (*v*. no)^c^	1.41 [1.21–1.61]	<0.001	1.18 [1.07–1.29]	0.001	1.20 [1.08–1.33]	0.002	1.01 [0.84–1.17]	0.96

a Odds ratios were derived from multinomial logistic regression analysis using a three-step approach in Mplus software. All the covariates presented in the table were entered into LGMM as auxiliary variables.

b Places severely affected by COVID-19 included places severely affected by initial peak and places with COVID-19 resurgences.

c COVID-19-related stressful life events included being COVID-19 patients, their family members or close contacts and being workers directly engaged in COVID-19 control or their family members.

### Evolution of psychopathological network among the general population after COVID-19 outbreak

**Fig. 4.**
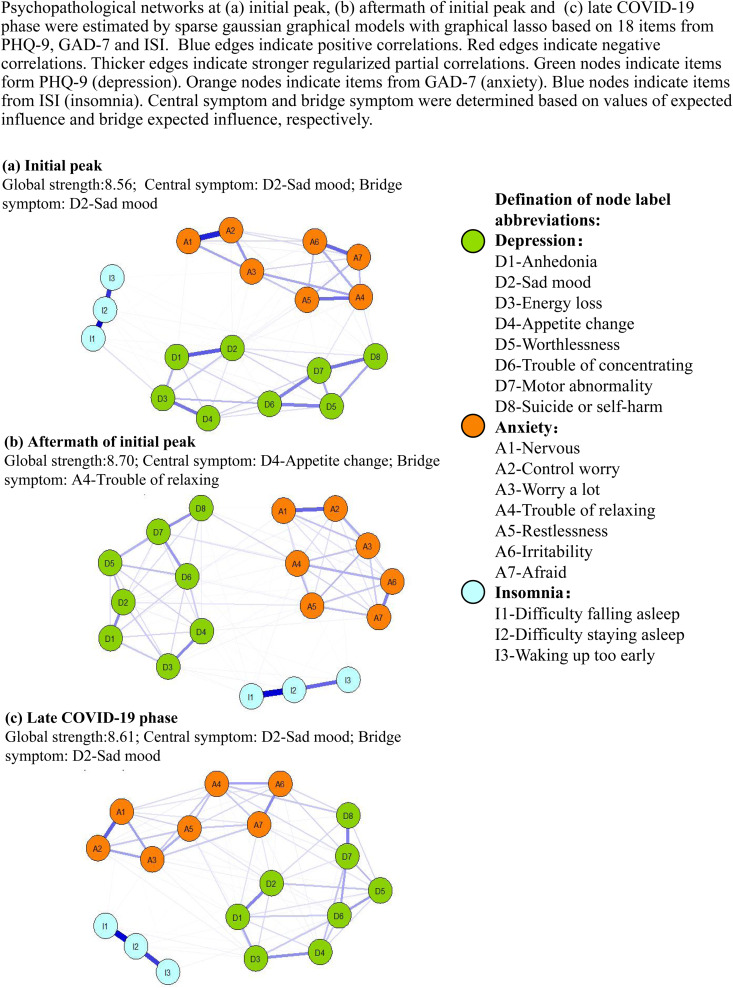
Evolution of psychopathological networks for depression, anxiety and insomnia after COVID-19. Psychopathological networks at (*a*) initial peak, (*b*) aftermath of initial peak and (*c*) late COVID-19 phase were estimated by sparse Gaussian graphical models with graphical lasso based on 18 items from PHQ-9, GAD-7 and ISI. Blue edges indicate positive correlations. Red edges indicate negative correlations. Thicker edges indicate stronger regularised partial correlations. Green nodes indicate items from PHQ-9 (depression). Orange nodes indicate items from GAD-7 (anxiety). Blue nodes indicate items from ISI (insomnia). Central symptom and bridge symptom were determined based on values of expected influence and bridge expected influence, respectively. (*a*) Initial peak: global strength: 8.56; central symptom: D2-Sad mood; bridge symptom: D2-Sad mood; (*b*) aftermath of initial peak: global strength: 8.70; central symptom: D4-Appetite change; bridge symptom: A4-Trouble of relaxing and (*c*) late COVID-19 phase: global strength: 8.61; central symptom: D2-Sad mood; bridge symptom: D2-Sad mood.

The global connectivity of psychopathological network significantly increased from initial COVID-19 peak to aftermath of initial peak (global strength at initial peak: 8.56; global strength in the aftermath of initial peak: 8.70; *p* < 0.001), then mildly decreased from aftermath of initial peak to late COVID-19 phase (global strength in the aftermath of initial peak: 8.70; global strength in the late COVID-19 phase: 8.61; *p* = 0.08). The global connectivity of psychological network at initial peak and late COVID-19 phase did not differ significantly (*p* = 0.19; Fig. 4; online Supplementary Fig. S2).

In the overall population, central and bridge symptoms were both ‘D2-Sad mood’ for initial peak and late COVID-19 phase (online Supplementary Figs S3 and S4). However, for the aftermath of initial peak, central symptom was ‘D4-Appetite change’ (online Supplementary Fig. S3), and bridge symptom was ‘A4-Trouble of relaxing’ (online Supplementary Fig. S4).

For individuals in ‘moderate/severe stable’ class, central symptoms for initial peak, aftermath of initial peak and late COVID-19 phase were ‘D7-Motor abnormality’, ‘A4-Trouble of relaxing’ and ‘D7-Motor abnormality’, respectively, while bridge symptoms for the three stages were ‘D7-Motor abnormality’, ‘D4-Appetite change’ and ‘A7-Afraid’, respectively (online Supplementary Figs S5–S7). For individuals following fluctuating psychological trajectories (‘mild-increase to decrease’ class and ‘mild-decrease to increase’ class), central or bridge symptoms were mainly ‘D2-Sad mood’ at all three pandemic phases (online Supplementary Figs S8–S10). For individuals in ‘mild stable’ class, central or bridge symptoms were mainly ‘D8-Suicide and self-harm’ and ‘D2-Sad mood’ (online Supplementary Figs S11–S13). For individuals in ‘normal stable’ class, central or bridge symptoms were mainly ‘A5-Restlessness’ and ‘D2-Sad mood’ (online Supplementary Figs S14–S16).

## Discussion

In this research, we found an overall ameliorating trend in mental health symptoms after COVID-19 outbreak. Five psychological symptom trajectory classes with different demographic and psychopathological characteristics were identified: normal stable (63.4%), mild stable (15.3%), mild-increase to decrease (11.7%), mild-decrease to increase (4.0%) and moderate/severe stable (5.5%). General population showed distinct psychopathological networks at different pandemic phases. Aftermath of initial peak may be a psychologically vulnerable period with specific psychopathological structures. To the best of our knowledge, this research is the first to provide a comprehensive long-term psychological profile of the general population after COVID-19 from a multidimensional perspective. We believe our findings are valuable for long-term population- and time-specific mental health management following COVID-19 and future pandemics.

The overall improving trend of mental health status after COVID-19 complies with findings from other countries, including the United Kingdom, the United States, Germany, Spain and India (Daly *et al*., 2020; Gopal *et al*., 2020; Bendau *et al*., 2021; Daly and Robinson, 2021). Improvement in pandemic control, lift of lockdown, removal of imperative measures and economic recovery may all contribute to the relief of mental strain (Daly *et al*., 2020; Gopal *et al*., 2020; Bendau *et al*., 2021; Daly and Robinson, 2021).

Despite the overall ameliorating trend in psychological symptoms, we found that there were still about 5% individuals showing persistently moderate-to-severe distress and approximately 16% following fluctuating psychological trajectories. The psychological symptom trajectory patterns identified in our research comply with findings from other countries, including the United Kingdom and Australia (Batterham *et al*., 2021; Pierce *et al*., 2021). We all found that although the majority of individuals showed consistently good mental health status, there were a small fraction of individuals showing persistently severe symptoms, and a considerable proportion of individuals beginning with mild symptoms, but following diverse trajectories afterwards. These findings suggest that apart from individuals showing severe distress, special attention should also be paid to those showing mild symptoms at initial peak, since their symptoms may fluctuate as the pandemic evolves.

In this research, some demographic factors, infection-related factors and post-COVID-19 repercussions were found to predict persistently severe psychological symptoms after COVID-19 outbreak. These predictors largely overlapped with those identified in other relevant studies (Daly *et al*., 2020; Fancourt *et al*., 2021). Young adults' long-term distress might be attributable to their higher exposure to social media and misinformation, more significant increases in work burden as well as greater insecurity in jobs and finance (Ganson *et al*., 2021). Individuals with low incomes or unmarried status might be less capable of coping with the financial adversities due to COVID-19 (Fancourt *et al*., 2021). COVID-19 patients, their family members and high-risk workers might experience substantial fears, witness traumatic events and bear overwhelming workloads, which could trigger enduring stress responses, emotional exhaustion and burn-outs (Lee *et al*., 2007; Iob *et al*., 2020; Mazza *et al*., 2020; Saghafi *et al*., 2021). The negative mental health impacts of local resurgences might be partially explained by the ‘double blows’ effects (Spittlehouse *et al*., 2014; Tang *et al*., 2017). Overwhelming workloads and unemployment, as common psychological stressors, might have magnified psychological effects in the background of pandemics due to the large-scale economic recession and life disturbances (McKee-Ryan *et al*., 2005; Maslach and Leiter, 2016; Shaw *et al*., 2020). Moreover, we identified that motor abnormality might be a potential treatment target symptom among individuals with persistently severe symptoms, complying with other research conducted during COVID-19 and indicating psychological benefits of physical exercises during lockdown (Wang *et al*., 2020).

Furthermore, we found that individuals following fluctuating psychological trajectories were more likely to report lower educational level and history of chronic diseases or psychiatric disorders. Low educational level and poor health status may impair psychological resilience, contributing to greater vulnerability to psychological stressors and instability of mental health status (Davydov *et al*., 2010; Wu *et al*., 2016; Xiao *et al*., 2019). Additionally, we identified ‘sad mood’ as the potential treatment target symptom among these individuals. We believe these findings can provide valuable information for psychological management during pandemics.

It is noteworthy that our study population showed highest network connectivity in the aftermath of initial peak among the three pandemic phases, indicating greatest susceptibility to mental disorders in the special phase. The findings suggest that aftermath of initial peak may be a psychologically vulnerable period, which is consistent with previous studies identifying increase in psychological symptoms at the end of lockdown (Saunders *et al*., 2021*a*, 2021*b*). One possible explanation is that during this period, most pandemic restrictions were removed and life largely came back to normal (e.g. large-scale return to work, economic recovery, increases in workloads), while the pandemic control situation was still unstable with emergence of sporadic cases and local resurgences (Shi *et al*., 2021*a*). The instability might arouse senses of uncertainty and insecurity, which in turn enhanced psychological vulnerability (Mohamed *et al*., 2021).

Additionally, central symptom (‘appetite change’) and bridge symptom (‘trouble of relaxing’) for aftermath of initial peak were totally different from the potential target symptoms (‘sad mood’) at the other two pandemic phases. The findings indicated that in the aftermath of initial peak, general population not only showed greater psychological vulnerability, but also had special psychopathological structures warranting different intervention strategies. The greater centrality of anxiety and somatic symptoms during this phase might be attributable to senses of uncertainty caused by social instability (Mohamed *et al*., 2021). Our findings indicate that special attention should be paid to this specific period. Intermittent emergence of sporadic cases, local resurgences and frequently changing pandemic control measures can lead to social instability witnessed by aftermath of initial peak, thus causing persistence of distress (Shi *et al*., 2021*a*). We believe the findings can provide implications for psychological management in future COVID-19 waves.

### Strengths and limitations

This research is the first to provide a comprehensive long-term psychological profile of general population following COVID-19 outbreak from a multidimensional perspective. The major strengths of this research included large and nationwide sample, as well as a relatively long observational period. Moreover, we provided in-depth information for population- and time-specific mental health management by incorporating multidimensional psychological modelling approaches.

However, this research has several limitations. First, due to online recruitment, our sample had a bias towards youngsters and highly educated people, and the follow-up rate was relatively low. The report rate for history of mental disorders was also low compared with other studies (Fancourt *et al*., 2021). It might be attributable to stigma towards mental illnesses and low recognition rate of mental health symptoms in China (Wang *et al*., 2019; Zhang *et al*., 2019; Fancourt *et al*., 2021), and bring bias to our results. Generalisation of our results to other populations should be made with caution. However, since the demographics did not differ much between our longitudinal sample and the baseline full sample, we believe the bias brought by drop-outs can be partially avoided (online Supplementary Table S6). Second, the observational duration might not be long enough to capture the complete trajectories. Third, mental health symptoms were evaluated with self-reported standardised questionnaires instead of clinical diagnoses. Fourth, we only considered three mental symptoms when exploring psychological symptom trajectory patterns and psychopathological networks, and the results have not been validated in other samples. Fifth, we only conducted three surveys after COVID-19 outbreak to capture psychological symptom development. Although the time points chosen were highly representative of different pandemic stages, limited number of time points might still preclude detailed depiction of trajectories. Finally, network analysis is rooted in the theory of ‘psychopathological network’ assuming mental disorders as networks based on causal interactions among individual symptoms. Since the theory lacks strong supporting evidence, our findings should be interpreted with caution, especially when guiding clinical practice (Bringmann *et al*., 2022). Therefore, future relevant research studies based on larger and more representative samples, adopting more objective measurements, involving multiple psychological symptoms and time points and conducted in other countries are called for to further validate our findings.

## Conclusions

This research provides a comprehensive and multidimensional long-term psychological profile of general population after COVID-19 outbreak. We drew three main conclusions: (1) mental health status generally improves among general population in 12 months after COVID-19; (2) there are still about 5% individuals showing persistently severe distress and approximately 16% following fluctuating psychological trajectories who demonstrate distinctive demographic characteristics and psychopathological structures. For individuals with persistently severe distress, the potential target symptom is ‘motor abnormality’, while for individuals following fluctuating trajectories, the potential target symptom is ‘sad mood’; (3) aftermath of initial COVID-19 peak may be a psychologically vulnerable period deserving special attention. ‘Sad mood’ emerges as a potential treatment target for initial peak and late COVID-19 phase. However, ‘appetite change’ and ‘trouble of relaxing’ emerge as potential treatment targets for aftermath of initial peak. We believe our findings can offer population- and time-specific reference for long-term mental health management after pandemics.

## Data Availability

The corresponding authors have full access to all the data in the study and take responsibility for the integrity of the data and the accuracy of the data analyses.

## Supplementary material

For supplementary material accompanying this paper visit http://doi.org/10.1017/S2045796022000518. click here to view supplementary material
