# Supplementary material for: Long-term psychological profile of general population following COVID-19 outbreak: symptom trajectories and evolution of psychopathological network
Source: Epidemiol Psychiatr Sci. 2022 Sep 27;31:e69. doi: 10.1017/S2045796022000518 (PMC9531590; doi:10.1017/S2045796022000518)
Supplement: Supplementary file 1 [file epssup.zip › S2045796022000518sup001.docx]

| **Table S1. Demographic and epidemic-related characteristics of the longitudinal sample.** | |
| --- | --- |
| **Factors** | **No. (%)/ Mean (SD)** |
| **Overall** | 18804 (100.0) |
| **Gender** |  |
| Male | 8558 (45.5) |
| Female | 10246 (54.5) |
| **Mean for age (SD)** | 36.6 (8.2) |
| **Age group (years)** |  |
| 18-39 | 12364 (65.8) |
| ≥ 40 | 6440 (34.2) |
| **Living area** |  |
| Urban | 17599 (93.6) |
| Rural | 1205 (6.4) |
| **Educational level** |  |
| College school or higher | 15489 (82.4) |
| Lower than college school | 3315 (17.6) |
| **Marital status** |  |
| Married | 14783 (78.6) |
| Unmarried | 4021 (21.4) |
| **Income level(yuan)** |  |
| 0-4999 | 4186 (22.3) |
| ≥5000 | 14618 (77.7) |
| **History of chronic diseases** |  |
| Yes | 1201 (6.4) |
| Unknown/no | 17603 (93.6) |
| **History of mental disorders** |  |
| Yes | 122 (0.6) |
| Unknown/no | 18682 (99.4) |
| **Family history of mental disorders** |  |
| Yes | 235 (1.2) |
| Unknown/no | 18569 (98.8) |

| **Table S2. Model fitting indicators for LGCMs for depression, anxiety and insomnia with different growth factors.** | | | | |
| --- | --- | --- | --- | --- |
| **Models** | **CFI** | **Chi-square** | **RMSEA** | **SRMR** |
| **Depression** | | | | |
| **Intercept only** | 0.969 | 274.135 | 0.015 | 0.015 |
| **Linear slope with fixed factor loadings** | 0.981 | 162.321 | 0.017 | 0.011 |
| **Linear slope with free factor loadings** | 0.992 | 79.493 | 0.011 | 0.007 |
| **Anxiety** | | | | |
| **Intercept only** | 0.901 | 682.939 | 0.025 | 0.023 |
| **Linear slope with fixed factor loadings** | 0.988 | 102.353 | 0.013 | 0.010 |
| **Linear slope with free factor loadings** | 0.986 | 132.63 | 0.015 | 0.010 |
| **Insomnia** | | | | |
| **Intercept only** | 0.971 | 402.820 | 0.028 | 0.015 |
| **Linear slope with fixed factor loadings** | 0.988 | 101.389 | 0.013 | 0.010 |
| **Linear slope with free factor loadings** | 0.989 | 138.645 | 0.016 | 0.010 |
| Note: CFI=Comparative fit index; SRMR=Standardized root-mean-square residual; RMSEA=Root mean square error of approximation. | | | | |

| **Table S3. Model fit statistics of LGMMs with 2-11 trajectory classes.** | | | | | | |
| --- | --- | --- | --- | --- | --- | --- |
| **Number of classes** | **AIC** | **BIC** | **aBIC** | **Entropy** | **VLMR-LRT p value** | **Proportion for each latent class (%)** |
| **2 classes** | 942710.094 | 942906.140 | 942826.691 | 0.989 | <0.001 | 90.5/9.5 |
| **3 classes** | 715565.281 | 715816.219 | 715714.525 | 0.933 | <0.001 | 72.9/18.1/9.0 |
| **4 classes** | 704280.852 | 704586.683 | 704462.743 | 0.921 | <0.001 | 66.3/17.1/8.7/7.9 |
| **5 classes** | **696252.150** | **696612.874** | **696466.688** | **0.921** | **<0.001** | **63.4/15.3/11.7/5.6/4.0** |
| **6 classes** | 690902.596 | 691318.213 | 691149.781 | 0.916 | 0.4601 | 63.3/12.9/10.9/5.7/4.5/2.7 |
| **7 classes** | 685596.679 | 686067.188 | 685876.511 | 0.918 | 0.0765 | 58.7/12.8/8.9/8.6/5.1/3.4/2.6 |
| **8 classes** | 681103.500 | 681628.902 | 681415.980 | 0.912 | 0.0702 | 58.4/12.6/8.6/7.7/4.7/3.2/2.6/1.3 |
| **9 classes** | 680926.566 | 681506.861 | 681271.692 | 0.904 | 0.3713 | 55.3/10.2/8.0/6.9/6.3/4.6/3.5/3.0/2.3 |
| **10 classes** | 675429.402 | 676064.590 | 675807.176 | 0.894 | 0.5354 | 57.1/10.3/7.8/6.6/4.6/3.9/3.7/3.3/1.7/0.9 |
| **11 classes** | 674644.470 | 675334.551 | 675054.892 | 0.889 | 0.5827 | 56.5/8.9/6.2/6.2/4.9/4.4/4.3/2.7/2.6/1.7/1.6 |
| Note: AIC = Akaike Information Criterion; BIC = Bayesian Information Criterion; VLMR-LRT = Vuong–Lo– Mendell –Rubin likelihood ratio test. | | | | | | |

| **Table S4. Predicted trajectories of latent symptom trajectory classes from the best-fitting 5-class LGMM.** | | | | | | |
| --- | --- | --- | --- | --- | --- | --- |
| **Latent symptom co-developmental trajectory classes** | **Depression (PHQ-9)** | | **Anxiety (GAD-7)** | | **Insomnia (ISI)** | |
|  | **Mean for intercept (SE)** | **Mean for slope**  **(SE)** | **Mean for intercept (SE)** | **Mean for slope**  **(SE)** | **Mean for intercept (SE)** | **Mean for slope**  **(SE)** |
| **Normal-stable** | 0.76 (0.02)*** | -0.17  (0.01)*** | 0.83  (0.03)*** | -0.24  (0.02)*** | 3.00  (0.03)*** | -0.16  (0.02)*** |
| **Mild-stable** | 6.98  (0.17)*** | 0.98  (0.09)*** | 5.79  (0.12)*** | 0.55  (0.07)*** | 8.81  (0.13)*** | 0.34  (0.08)*** |
| **Mild-increase to decrease** | 8.22  (0.22)*** | -4.05  (0.26)*** | 6.93  (0.18)*** | -3.27 (0.21)*** | 8.93  (0.18)*** | -2.93 (0.19)*** |
| **Mild-decrease to increase** | 8.46  (0.35)*** | 7.01  (0.40)*** | 6.95  (0.26)*** | 5.17 (0.32)*** | 7.80  (0.27)***, | 3.15  (0.26)*** |
| **Moderate to severe-stable** | 15.73  (0.25)*** | 0.06  (0.16) | 12.41  (0.21)*** | -0.13 (0.14) | 13.64  (0.19)*** | -0.65  (0.14)*** |
| Note：*** p < 0.001. | | | | | | |

| **Table S5. Demographic and epidemic-related characteristics of the five psychological symptom trajectory classes.** | | | | | |
| --- | --- | --- | --- | --- | --- |
| **Factors** | **Moderate/severe stable (n=1044)** | **Mild stable (n=2885)** | **Mild-increase to decrease (n=2201)** | **Mild-decrease to increase (n=755)** | **Normal stable (n=11919)** |
| **Gender** |  |  |  |  |  |
| Male | 611 (58.5) ^a,b,c,d^ | 1410 (48.9) ^d^ | 1126 (51.2) ^d^ | 355 (47.0) ^d^ | 5056 (42.4) |
| Female | 433 (41.5) | 1475 (51.1) | 1075 (48.8) | 400 (53.0) | 6863 (57.6) |
| **Age group (years)** |  |  |  |  |  |
| 18-39 | 789 (75.6) ^a,b,c,d^ | 2032 (70.4) ^d^ | 1533 (69.7) ^d^ | 527 (69.8) ^d^ | 7483 (62.8) |
| ≥ 40 | 255 (24.4) | 853 (29.6) | 668 (30.3) | 228 (30.2) | 4436 (37.2) |
| **Living area** |  |  |  |  |  |
| Urban | 955 (91.5) ^a,d^ | 2705 (93.8) | 2033 (92.4) ^d^ | 697 (92.3) | 11209 (94.0) |
| Rural | 89 (8.5) | 180 (6.2) | 168 (7.6) | 58 (7.7) | 710 (6.0) |
| **Educational level** |  |  |  |  |  |
| Lower than college school | 236 (22.6) ^a,b,d^ | 434 (15.0) ^c,d^ | 366 (16.6) | 142 (18.8) | 2137 (17.9) |
| College school or higher | 808 (77.4) | 2451 (85.0) | 1835 (83.4) | 613 (81.2) | 9782 (82.1) |
| **Marital status** |  |  |  |  |  |
| Married | 726 (69.5) ^a,b,c,d^ | 2160 (74.9) ^c,d^ | 1661 (75.5) ^c,d^ | 601 (79.6) | 9635 (80.8) |
| Unmarried | 318 (30.5) | 725 (25.1) | 540 (24.5) | 154 (20.4) | 2284 (19.2) |
| **Family income level (yuan)** |  |  |  |  |  |
| 0-4999 | 301 (28.8) ^a,b,c,d^ | 711 (24.6) ^d^ | 505 (22.9) ^d^ | 183 (24.2) ^d^ | 2486 (20.9) |
| ≥5000 | 743 (71.2) | 2174 (75.4) | 1696 (77.1) | 572 (75.8) | 9433 (79.1) |
| **History of chronic diseases** |  |  |  |  |  |
| Yes | 83 (8.0) ^d^ | 194 (6.7) | 151 (6.9) | 53 (7.0) | 720 (6.0) |
| No/unknown | 961 (92.0) | 2691 (93.3) | 2050 (93.1) | 702 (93.0) | 11199 (94.0) |
| **History of psychiatric disorders** |  |  |  |  |  |
| Yes | 29 (2.8) ^a,b,c,d^ | 31 (1.1) ^d^ | 29 (1.3) ^d^ | 10 (1.3) ^d^ | 23 (0.2) |
| No/unknown | 1015 (97.2) | 2854 (98.9) | 2172 (98.7) | 745 (98.7) | 11896 (99.8) |
| **Living in places severely affected by initial peak $** |  |  |  |  |  |
| Yes | 117 (11.2) ^a,b,c,d^ | 159 (5.5) ^b,d^ | 157 (7.1) ^d^ | 45 (6.0) ^d^ | 451 (3.8) |
| No | 927 (88.8) | 2726 (94.5) | 2044 (92.9) | 710 (94.0) | 11468 (96.2) |
| **Quarantine** |  |  |  |  |  |
| Yes | 540 (51.7) ^a,b,c,d^ | 1061 (36.8) ^b,c,d^ | 890 (40.4) ^d^ | 325 (43.0) ^d^ | 3564 (29.9) |
| No | 504 (48.3) | 1824 (63.2) | 1311 (59.6) | 430 (57.0) | 8355 (70.1) |
| **COVID-19 related stressful life events #** |  |  |  |  |  |
| Yes | 586 (56.1) ^a,b,c,d^ | 1237 (42.9) ^d^ | 971 (44.1) ^d^ | 347 (46.0) ^d^ | 4013 (33.7) |
| No | 458 (43.9) | 1648 (57.1) | 1230 (55.9) | 408 (54.0) | 7906 (66.3) |
| ^a^ p < 0.05 for chi-squared tests for proportion differences compared with the “Mild-stable " class. | | | | | |
| ^b^ p < 0.05 for chi-squared tests for proportion differences compared with the “Mild-increase to decrease" class. | | | | | |
| ^c^ p < 0.05 for chi-squared tests for proportion differences compared with the “Mild-decrease to increase" class. | | | | | |
| ^d^ p < 0.05 for chi-squared tests for proportion differences compared with the “Normal stable" class. | | | | | |
| $ Places severely affected by COVID-19 included places severely affected by initial peak and places with COVID-19 resurgences. | | | | | |
| #COVID-19 related stressful life events included being COVID-19 patients, their family members or close contacts, and being frontline workers or their family members. | | | | | |
|  | | | | | |

| **Table S6. Demographic characteristics of full baseline sample and longitudinal sample.** | | |
| --- | --- | --- |
| **Factors** | **Full baseline sample (n=56679)** | **Longitudinal sample (n = 18804)** |
| **Gender** | | |
| Male | 27149(47.9) | 8558(45.5) |
| Female | 29530(52.1) | 10246(54.5) |
| **Age group (years)** |  |  |
| 18-39 | 39468(69.6) | 12364(65.8) |
| ≥40 | 17211(30.4) | 6440(34.2) |
| **Living area** | | |
| Urban | 52839(93.2) | 17599(93.6) |
| Rural | 3840(6.8) | 1205(6.4) |
| **Educational level** | | |
| College school or higher | 47139(83.2) | 15489(82.4) |
| Lower than college school | 9540(16.8) | 3315(17.6) |
| **Marital status** | | |
| Married | 43763(77.2) | 14783(78.6) |
| Unmarried | 12916(22.8) | 4021(21.4) |
| **Family monthly income (yuan)** | | |
| 0-4999 | 13016(23.0) | 4186(22.3) |
| ≥5000 | 43663(77.0) | 14618(77.7) |
| **History of chronic diseases** | | |
| Yes | 3274(5.8) | 1201(6.4) |
| No or unknown | 53405(94.2) | 17603(93.6) |
| **History of psychiatric disorders** | | |
| Yes | 161(0.3) | 122(0.6) |
| No or unknown | 56518(99.7) | 18682(99.4) |
| **Family history of psychiatric disorders** | | |
| Yes | 396(0.7) | 235(1.2) |
| No or unknown | 56283(99.3) | 18569(98.8) |
