# Supplementary material for: Long-term psychological profile of general population following COVID-19 outbreak: symptom trajectories and evolution of psychopathological network
Source: Epidemiol Psychiatr Sci. 2022 Sep 27;31:e69. doi: 10.1017/S2045796022000518 (PMC9531590; doi:10.1017/S2045796022000518)

Fig.S1. Predicted trajectories of depression, anxiety and insomnia stratified by significant predictors.

All trajectories were depicted based on adjusted estimated means derived from conditional linear slope LGCMs . All models were adjusted for gender, age, educational level, living area, marital status, monthly family income, history of chronic diseases, history of psychiatric disorders, family history of psychiatric disorders, history of sleep disturbance, history of smoking , history of alcohol abuse, COVID-19 patients, family members of COVID-19 patients, close contacts of COVID-19 patients, engagement in COVID-19 related work, family members of workers directly engaging in COVID-19 related work, occupational exposure risk to COVID-19, quarantine, living in places severely affected by initial peak, local resurgences, increases in workloads, unemployment due to COVID-19, seeking psychological consultations, wearing face masks and reducing social gatherings.

Note: DEP = Depression; ANX = Anxiety; INS = Insomnia.

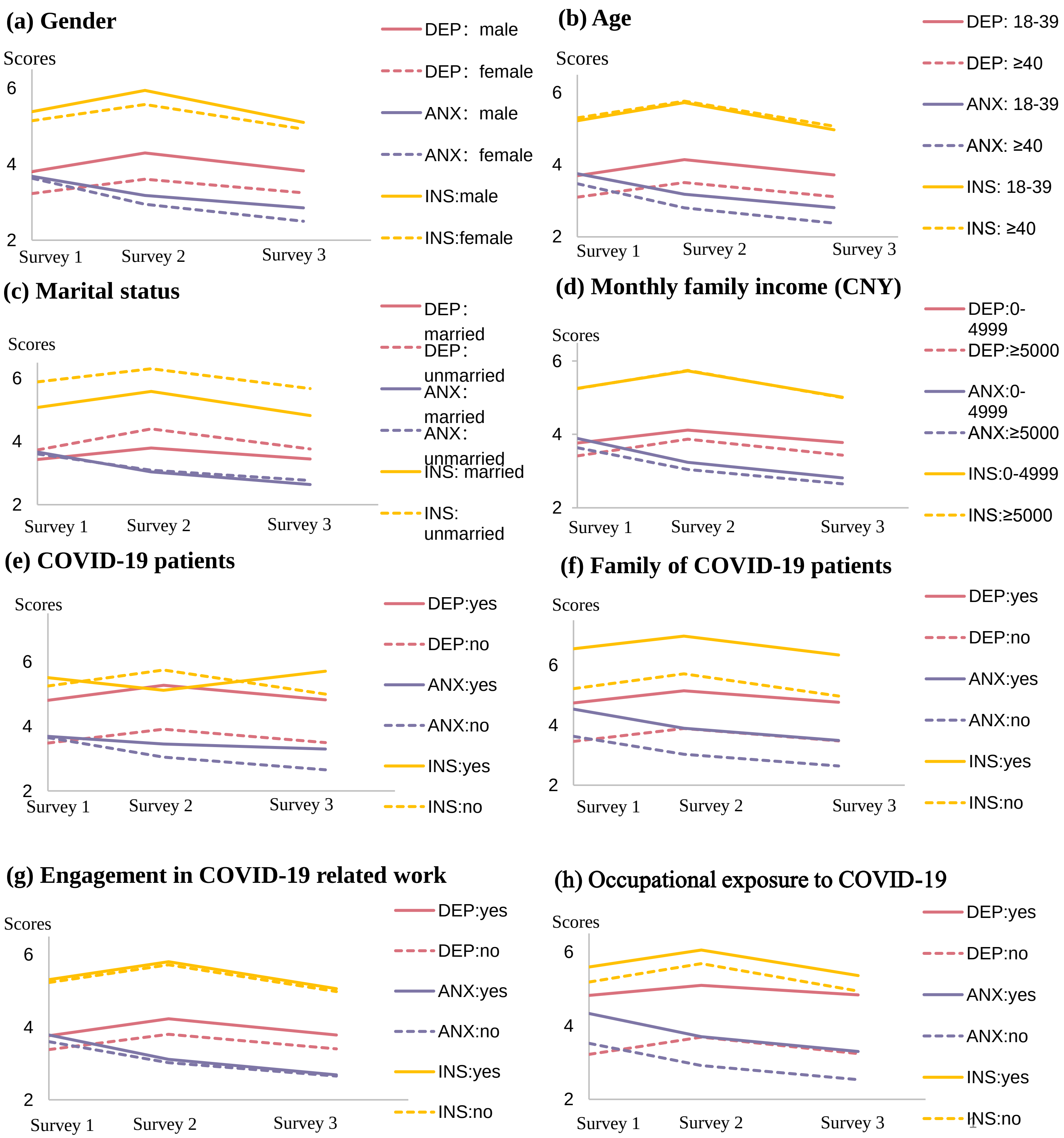

(Continued)

(i) Quarantine

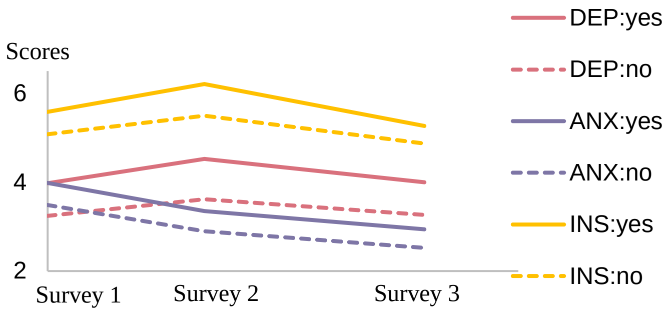

(g) Living in places severely affected by initial peak

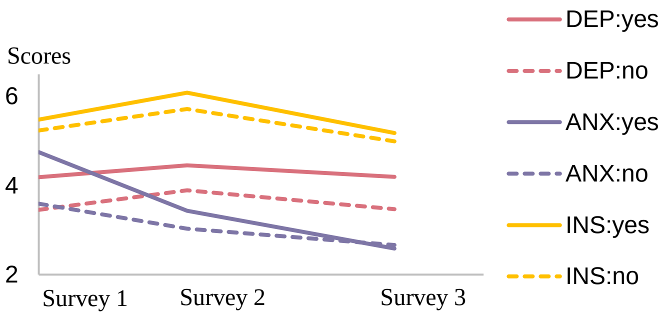

(k) Local resurgences

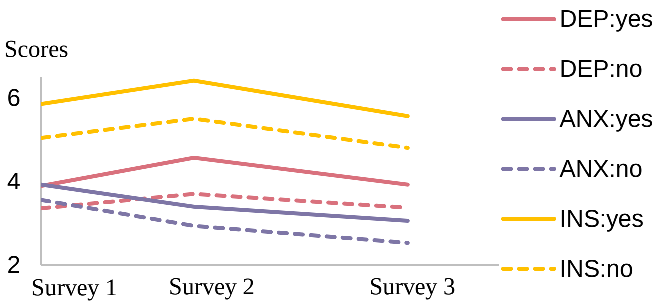

(l) Increases in workloads due to COVID-19

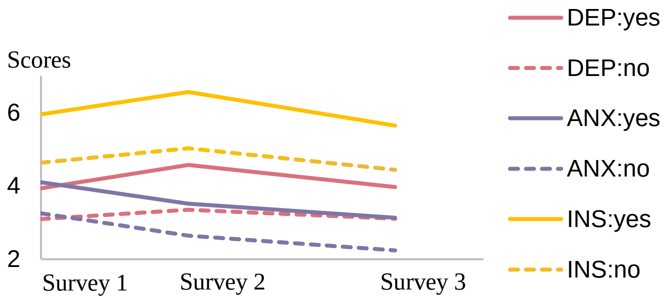

(m) Unemployment due to COVID-19

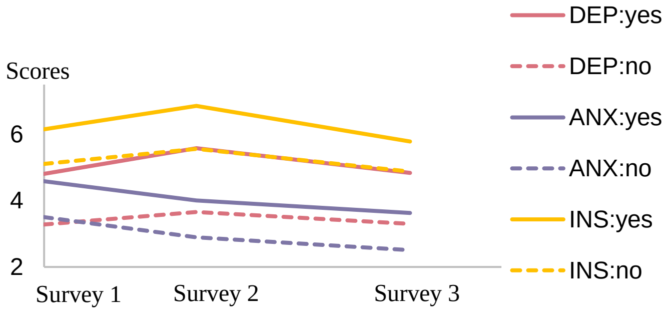

(n) Wearing facemasks

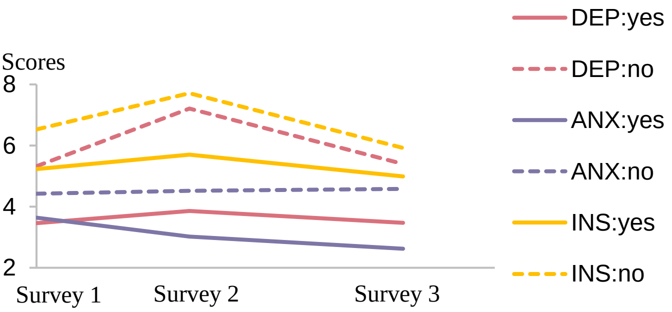

(o) Reducing social gatherings

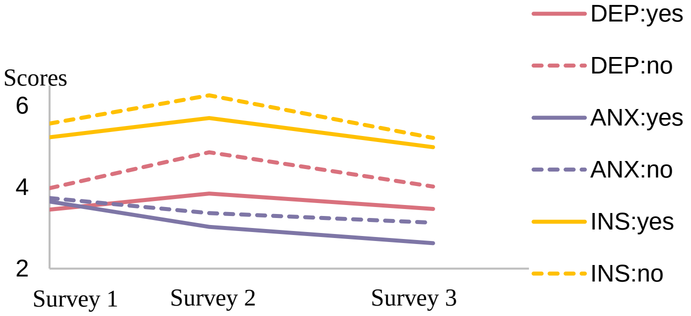

Fig. S2. Stability of psychopathological networks at (a) initial peak, (b) aftermath of initial peak and (c) late COVID-19 phase.
The x axle indicates the proportion of cases included, and the y axle indicates the correlations of strength with the original sample after dropping off a proportion of cases. Shades indicate 95% confidence intervals. The centrality stability (CS) coefficient is defined as the maximum drop proportions to retain correlation of 0.7 in at least 95% of the samples, which is calculated by R package “bootnet”. CS coefficient should be above 0.5 to consider the network as stable.

(a) Initial peak
CS coefficient: 0.93.

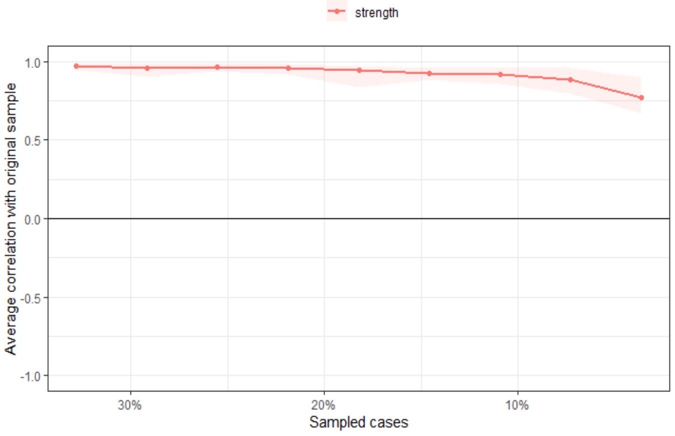

(b) Aftermath of initial peak
CS Coefficient: 0.88.

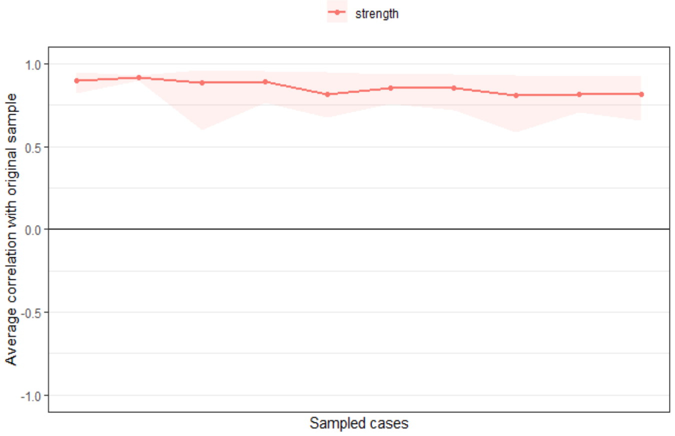

(c) Late COVID-19 phase
CS coefficient:0.85.

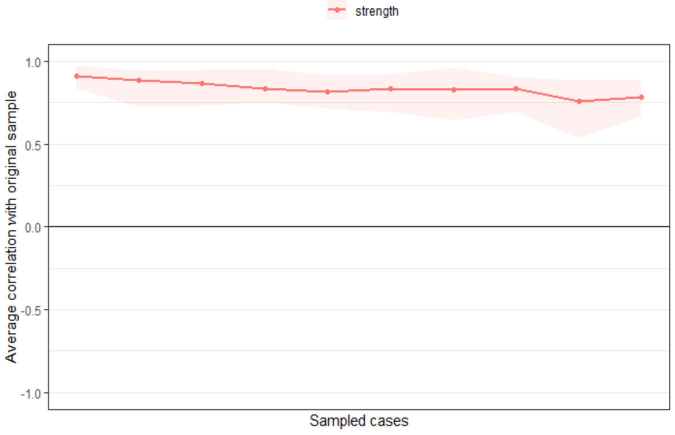

Fig. S3. Network centrality indices for psychopathological networks at (a) initial peak, (b) aftermath of initial peak and (c) late COVID-19 phase.

The nodes are ranked in descending order for values of expected influence.

(a) Initial peak

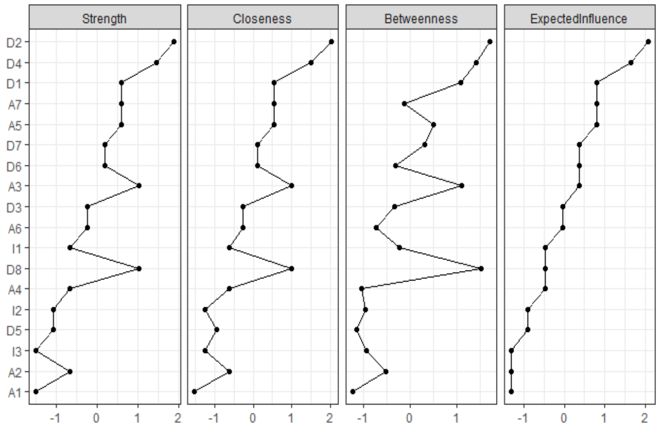

(b) Aftermath of initial peak

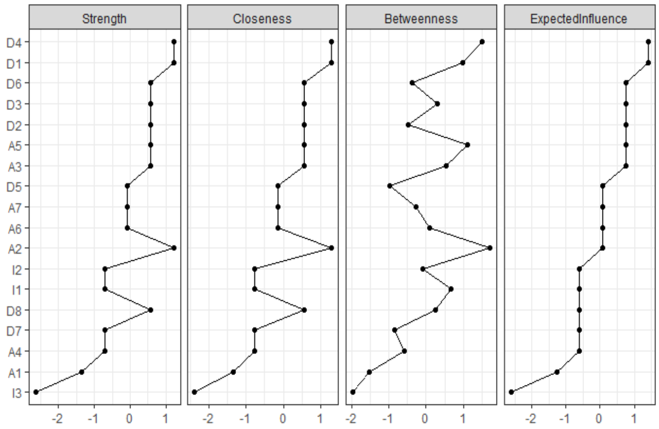

(c) Late COVID-19 phase

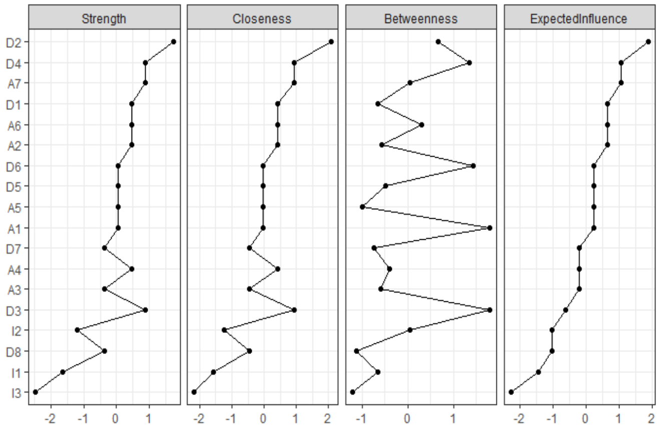

Fig. S4. Bridge centrality indices for psychopathological networks at (a) initial peak, (b) aftermath of initial peak and (c) late COVID-19 phase.

The nodes are ranked in descending order for bridge centrality indices.

(a) Initial peak

Bridge Strength | Bridge Betweenness | Bridge Closeness | Bridge Expected Influence

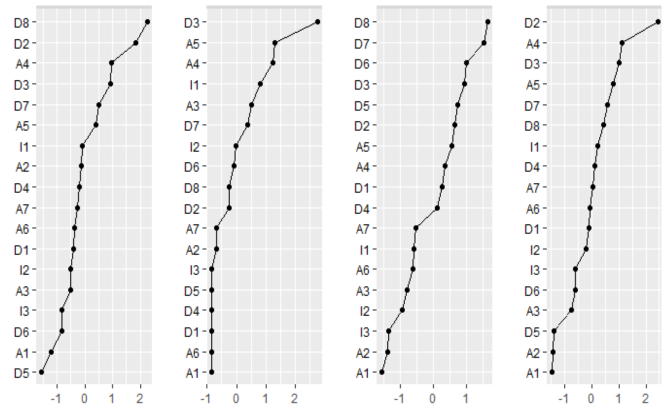

(b) Aftermath of initial peak

Bridge Strength | Bridge Betweenness | Bridge Closeness | Bridge Expected Influence

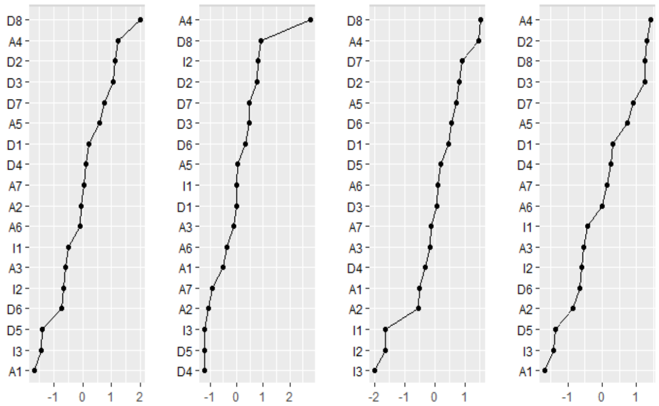

(c) Late COVID-19 phase

Bridge Strength | Bridge Betweenness | Bridge Closeness | Bridge Expected Influence

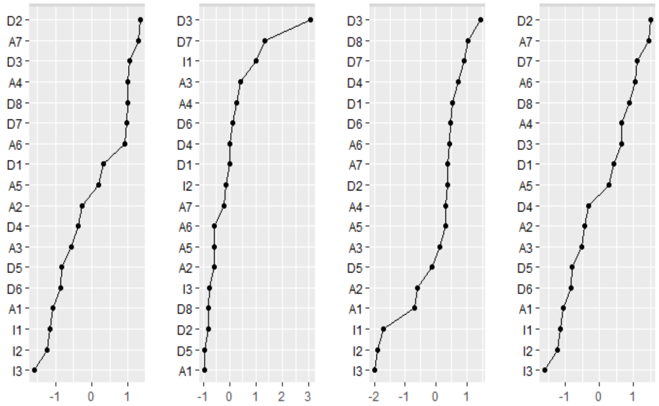

Fig. S5. Evolution of psychopathological network at (a) initial peak, (b) aftermath of initial peak and (c) late COVID-19 phase among individuals in "moderate/severe stable" trajectory class.

(a) Initial peak
Global strength:8.21; Central symptom: D7-Motor abnormality;
Bridge symptom: D7-Motor abnormality

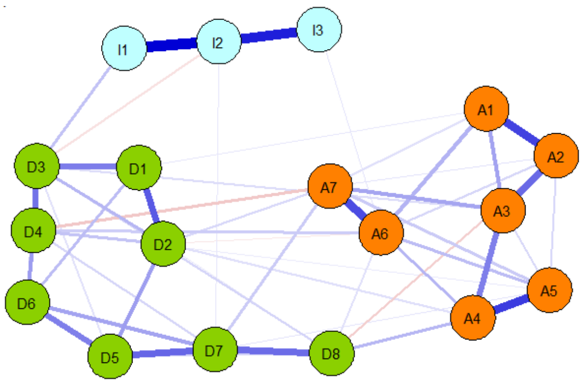

(b) Aftermath of initial peak
Global strength:6.69; Central symptom: A4-Trouble of relaxing; Bridge symptom: D4-Appetite change

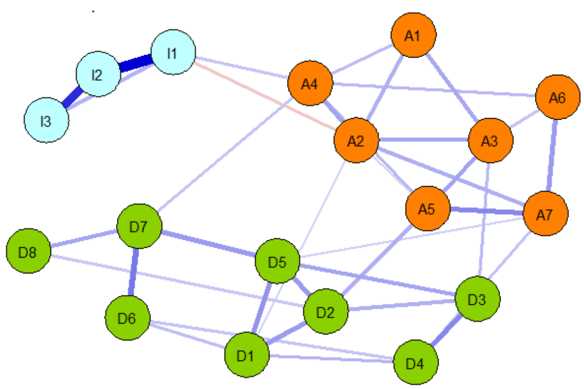

(c) Late COVID-19 phase
Global strength:5.30; Central symptom: D7-Motor abnormality;
Bridge symptom: A7-Afraid

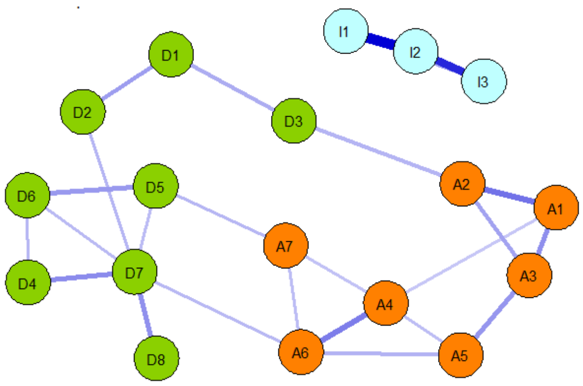

Defination of node label abbreviations:

Depression:
D1-Anhedonia
D2-Sad mood
D3-Energy loss
D4-Appetite change
D5-Worthlessness
D6-Trouble of concentrating
D7-Motor abnormality
D8-Suicide or self-harm

Anxiety:
A1-Nervous
A2-Control worry
A3-Worry a lot
A4-Trouble of relaxing
A5-Restlessness
A6-Irritability
A7-Afraid

Insomnia:
I1-Difficulty falling asleep
I2-Difficulty staying asleep
I3-Waking up too early

Fig. S6. Network centrality indices for psychopathological networks at (a) initial peak, (b) aftermath of initial peak and (c) late COVID-19 phase among individuals in "moderate/severe stable" trajectory class.

The nodes are ranked in descending order for values of expected influence.

(a) Initial peak

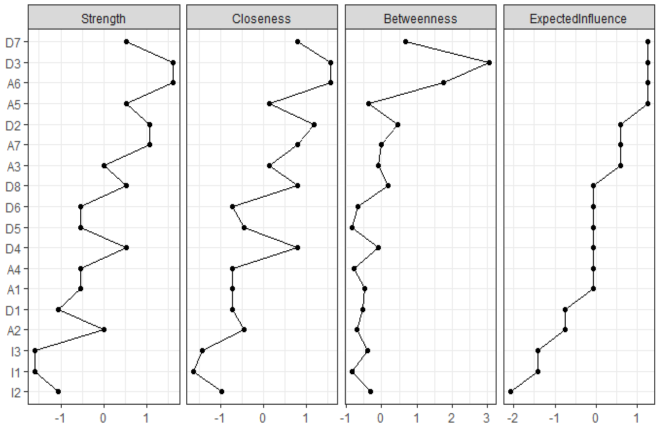

(b) Aftermath of initial peak

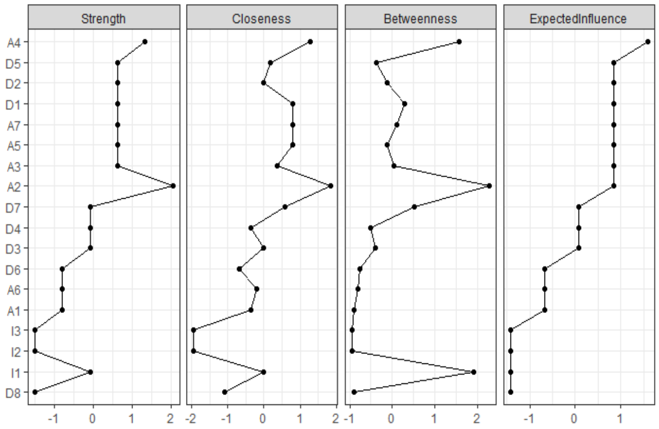

(c) Late COVID-19 phase

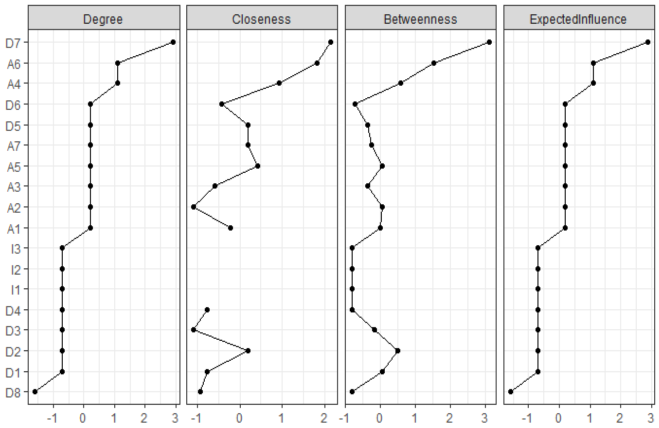

Fig. S7. Bridge centrality indices for psychopathological networks at (a) initial peak, (b) aftermath of initial peak and (c) late COVID-19 phase among individuals in “moderate/severe stable” trajectory class.

The nodes are ranked in descending order for bridge centrality indices.

(a) Initial peak

Bridge Strength | Bridge Betweenness | Bridge Closeness | Bridge Expected Influence

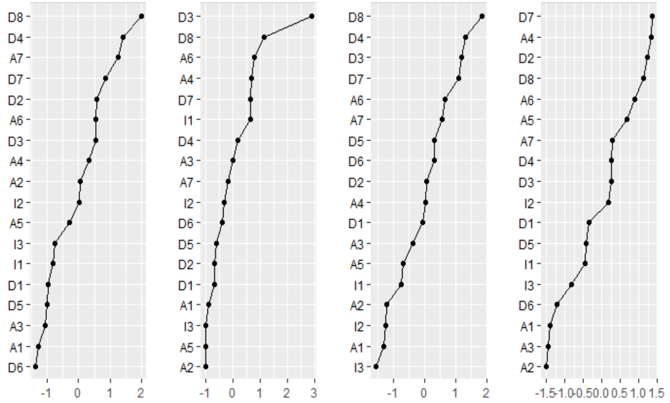

(b) Aftermath of initial peak

Bridge Strength | Bridge Betweenness | Bridge Closeness | Bridge Expected Influence

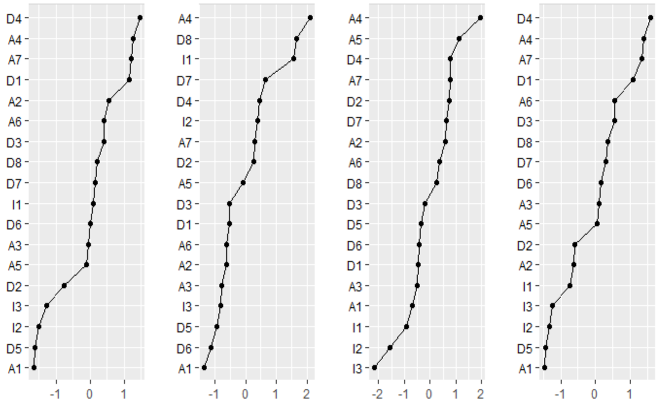

(c) Late COVID-19 phase

Bridge Strength | Bridge Betweenness | Bridge Closeness | Bridge Expected Influence

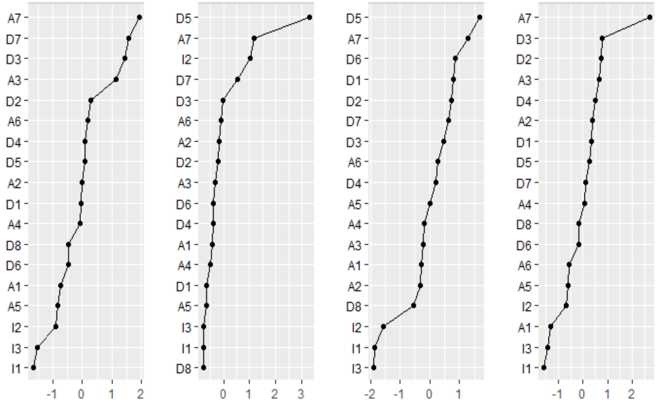

Fig. S8. Evolution of psychopathological network at (a) initial peak , (b) aftermath of initial peak and (c) late COVID-19 phase among individuals following fluctuating trajectories.

(a) Initial peak
Global strength:8.39; Central symptom: D2-Sad mood; Bridge symptom: D2-Sad mood

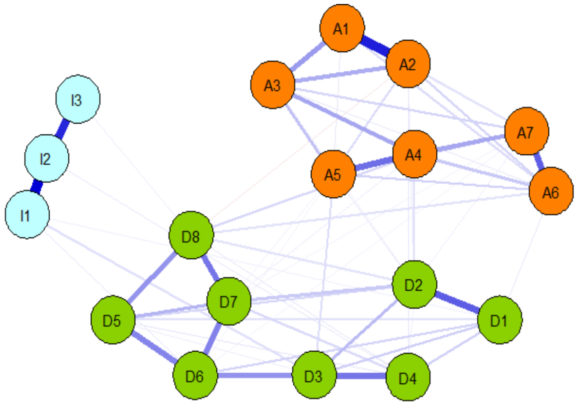

(b) Aftermath of initial peak
Global strength:8.26; Central symptom: D2-Sad mood; Bridge symptom: D2-Sad mood

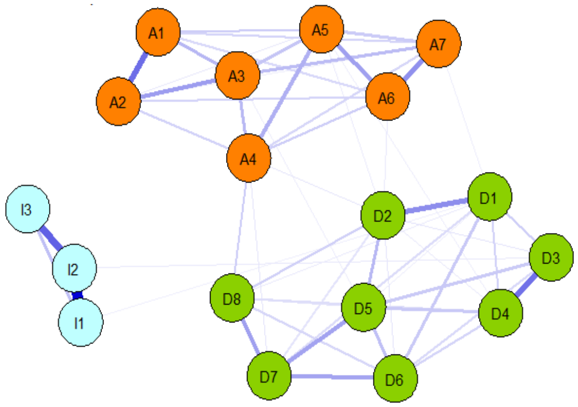

(c) Late COVID-19 phase
Global strength:8.65; Central symptom: A7-Afraid; Bridge symptom: D2-Sad mood

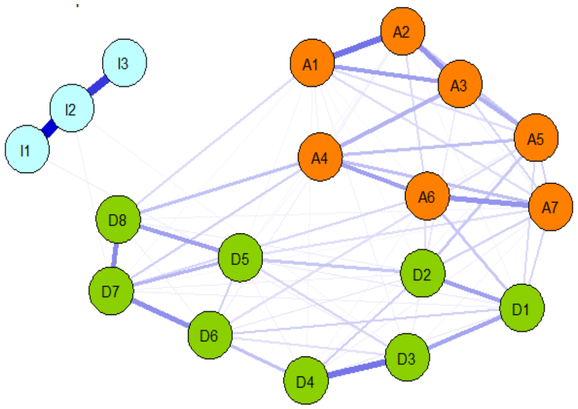

Defination of node label abbreviations:

Depression:
D1-Anhedonia
D2-Sad mood
D3-Energy loss
D4-Appetite change
D5-Worthlessness
D6-Trouble of concentrating
D7-Motor abnormality
D8-Suicide or self-harm

Anxiety:
A1-Nervous
A2-Control worry
A3-Worry a lot
A4-Trouble of relaxing
A5-Restlessness
A6-Irritability
A7-Afraid

Insomnia:
I1-Difficulty falling asleep
I2-Difficulty staying asleep
I3-Waking up too early

Fig. S9. Network centrality indices for psychopathological networks at (a) initial peak , (b) aftermath of initial peak and (c) late COVID-19 phase among individuals following fluctuating trajectories.

The nodes are ranked in descending order for values of expected influence.

(a) Initial peak

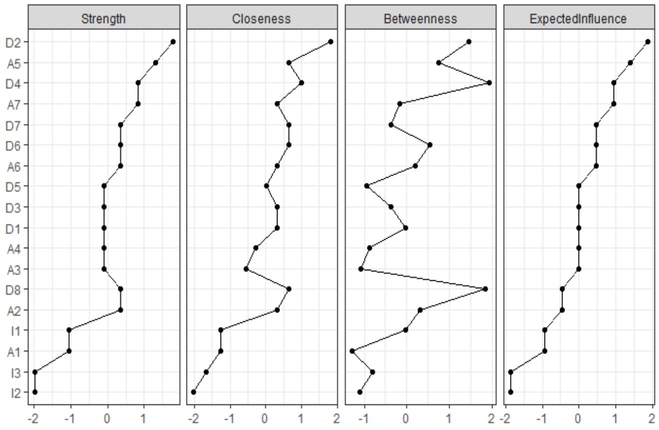

(b) Aftermath of initial peak

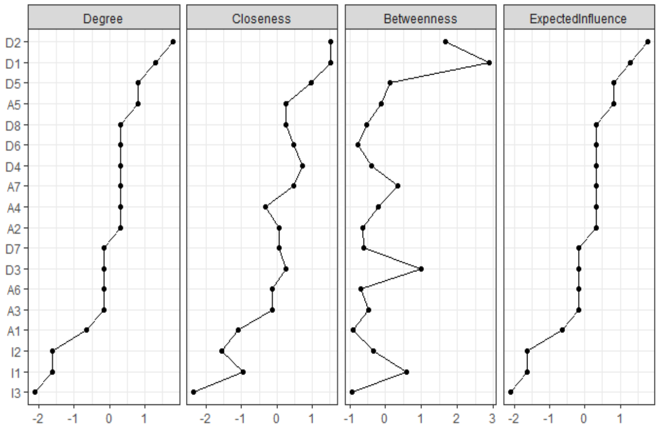

(c) Late COVID-19 phase

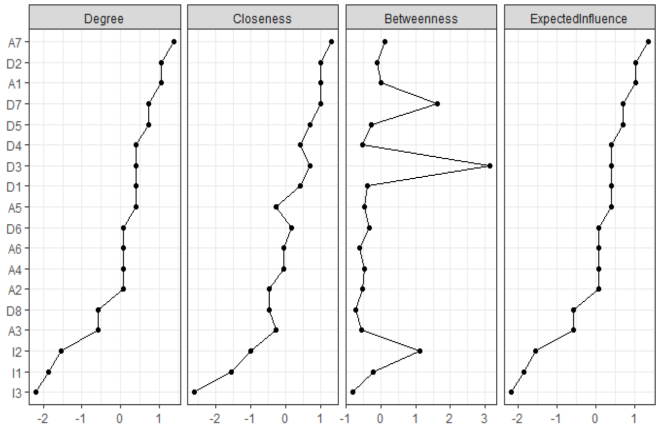

Fig. S10. Bridge centrality indices for psychopathological networks at (a) initial peak , (b) aftermath of initial peak and (c) late COVID-19 phase among individuals following fluctuating trajectories. The nodes are ranked in descending order for bridge centrality indices.

(a) Initial peak

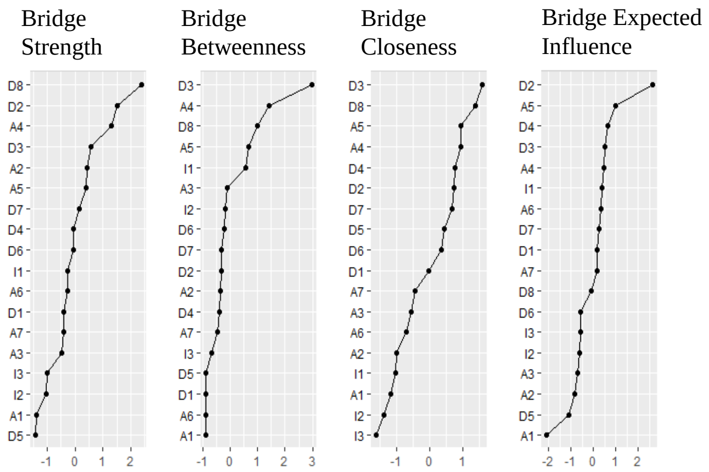

(b) Aftermath of initial peak

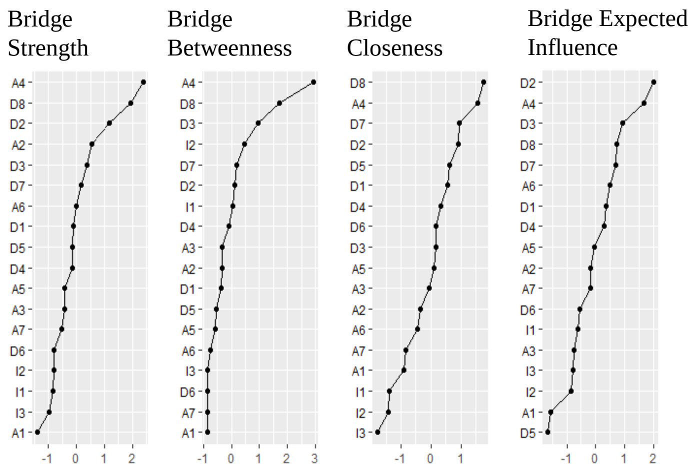

(c) Late COVID-19 phase

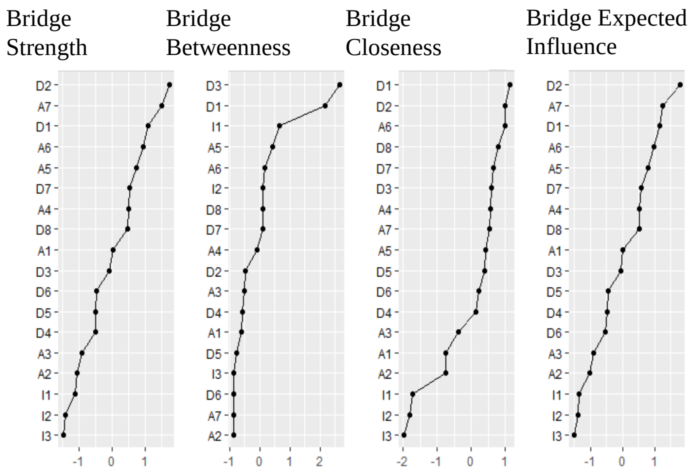

Fig. S11. Evolution of psychopathological network at (a) initial peak , (b) aftermath of initial peak and (c) late COVID-19 phase among individuals in "mild stable" trajectory class.

(a) Initial peak

Global strength:8.41; Central symptom: D2-Sad mood; Bridge symptom: D2-Sad mood

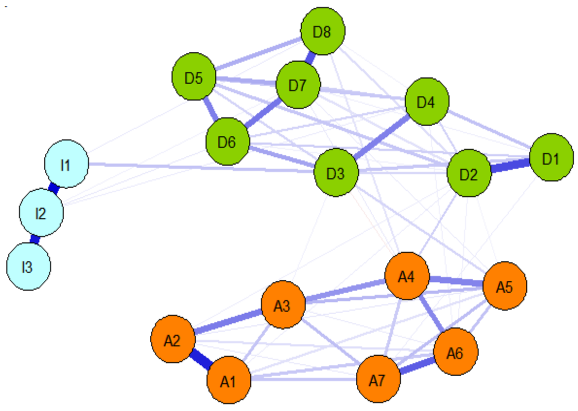

(b) Aftermath of initial peak

Global strength:7.85; Central symptom: A7-Afraid; Bridge symptom: D8-Suicide or self-harm

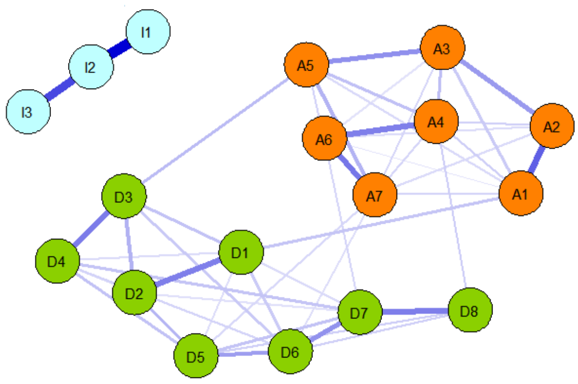

(c) Late COVID-19 phase

Global strength:5.71; Central symptom: D8-Suicide or self-harm; Bridge symptom: D8-Suicide or self-harm

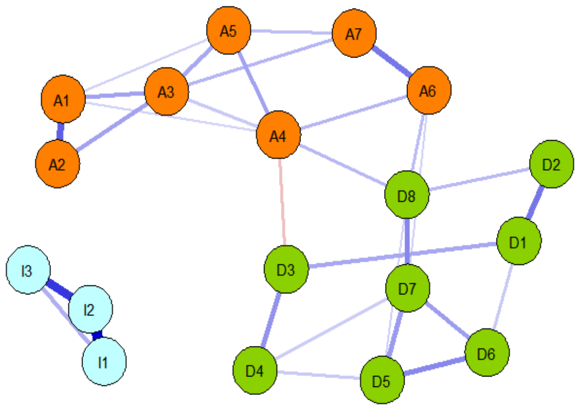

Defination of node label abbreviations:

Depression:
D1-Anhedonia
D2-Sad mood
D3-Energy loss
D4-Appetite change
D5-Worthlessness
D6-Trouble of concentrating
D7-Motor abnormality
D8-Suicide or self-harm

Anxiety:
A1-Nervous
A2-Control worry
A3-Worry a lot
A4-Trouble of relaxing
A5-Restlessness
A6-Irritability
A7-Afraid

Insomnia:
I1-Difficulty falling asleep
I2-Difficulty staying asleep
I3-Waking up too early

Fig. S12. Network centrality indices for psychopathological networks at (a) initial peak , (b) aftermath of initial peak and (c) late COVID-19 phase among individuals in “mild stable” trajectory class .

The nodes are ranked in descending order for values of expected influence.

(a) Initial peak

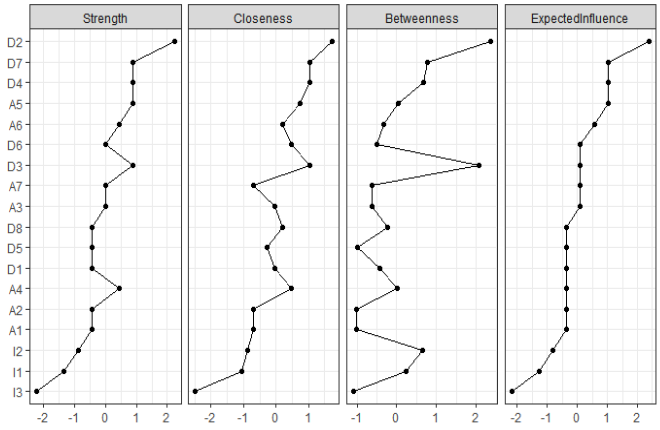

(b) Aftermath of initial peak

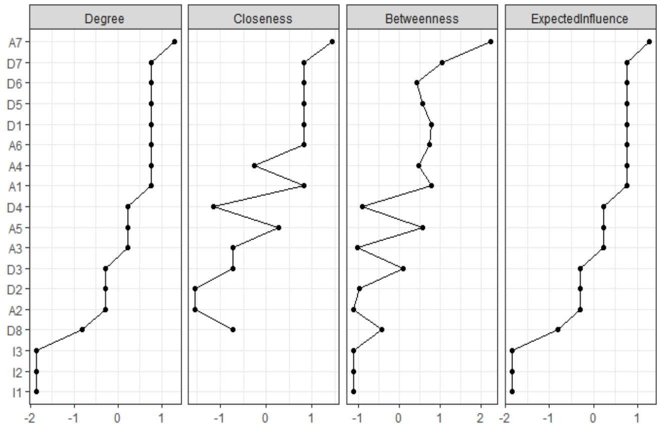

(c) Late COVID-19 phase

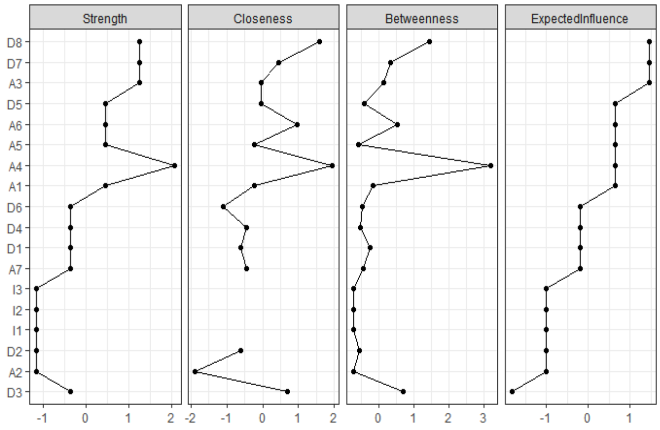

Fig. S13. Bridge centrality indices for psychopathological networks at (a) initial peak , (b) aftermath of initial peak and (c) late COVID-19 phase among individuals in “mild stable” trajectory class . The nodes are ranked in descending order for bridge centrality indices.

(a) Initial peak

Bridge Strength | Bridge Betweenness | Bridge Closeness | Bridge Expected Influence

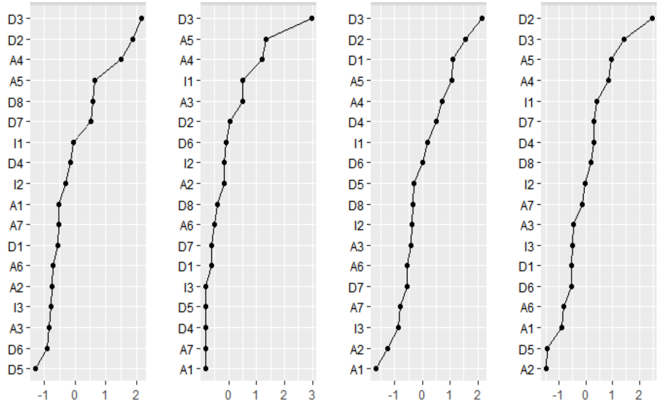

(b) Aftermath of initial peak

Bridge Strength | Bridge Betweenness | Bridge Closeness | Bridge Expected Influence

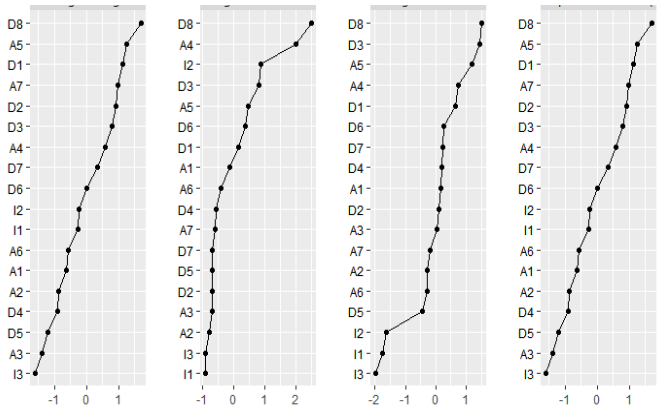

(c) Late COVID-19 phase

Bridge Strength | Bridge Betweenness | Bridge Closeness | Bridge Expected Influence

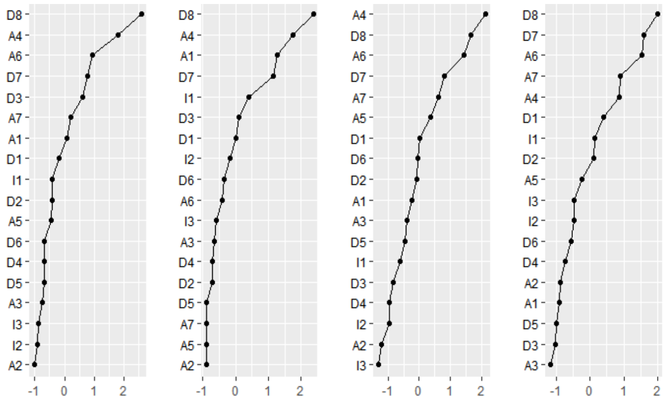

Fig. S14. Evolution of psychopathological network at (a) initial peak , (b) aftermath of initial peak and (c) late COVID-19 phase among individuals in "normal stable" trajectory class.

(a) Initial peak

Global strength:8.58; Central symptom: D2-Sad mood; Bridge symptom: D2-Sad mood

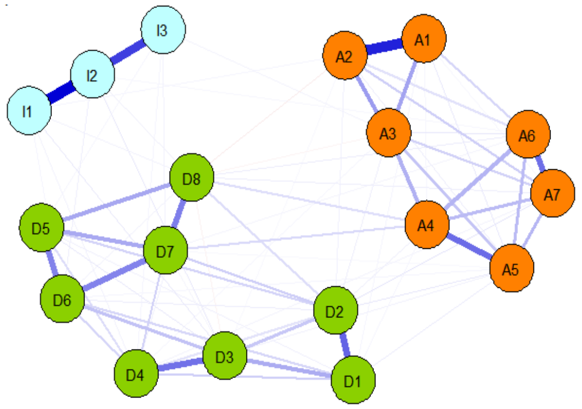

(b) Aftermath of initial peak

Global strength:7.65; Central symptom: A5-Restlessness; Bridge symptom: D3-Energy loss

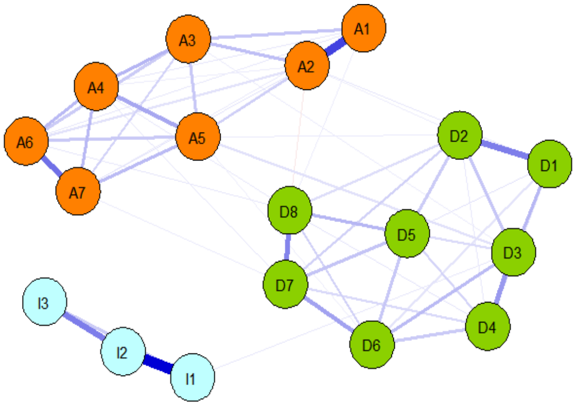

(c) Late COVID-19 phase

Global strength:7.07; Central symptom: A5-Restlessness; Bridge symptom: D2-Sad mood

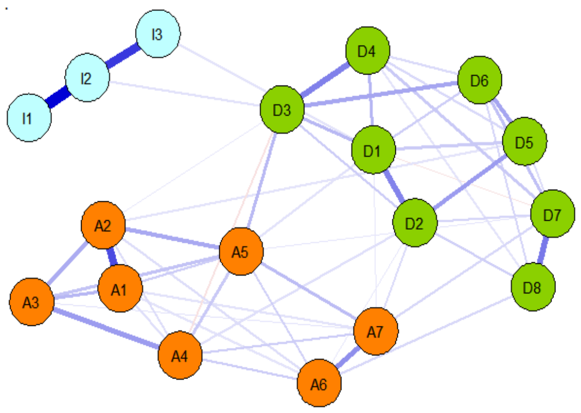

Defination of node label abbreviations:

Depression:

D1-Anhedonia
D2-Sad mood
D3-Energy loss
D4-Appetite change
D5-Worthlessness
D6-Trouble of concentrating
D7-Motor abnormality
D8-Suicide or self-harm

Anxiety:

A1-Nervous
A2-Control worry
A3-Worry a lot
A4-Trouble of relaxing
A5-Restlessness
A6-Irritability
A7-Afraid

Insomnia:

I1-Difficulty falling asleep
I2-Difficulty staying asleep
I3-Waking up too early

Fig. S15. Network centrality indices for psychopathological networks at (a) initial peak , (b) aftermath of initial peak and (c) late COVID-19 phase among individuals in “normal stable” trajectory class .

The nodes are ranked in descending order for values of expected influence.

(a) Initial peak

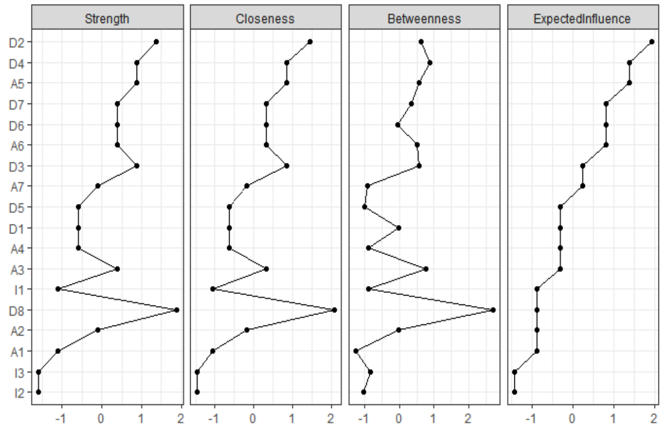

(b) Aftermath of initial peak

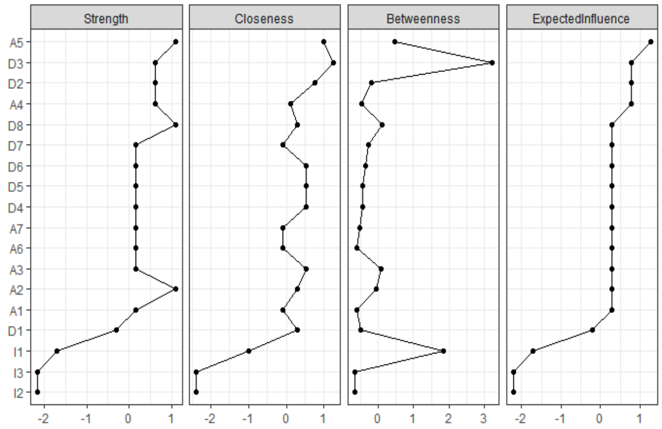

(c) Late COVID-19 phase

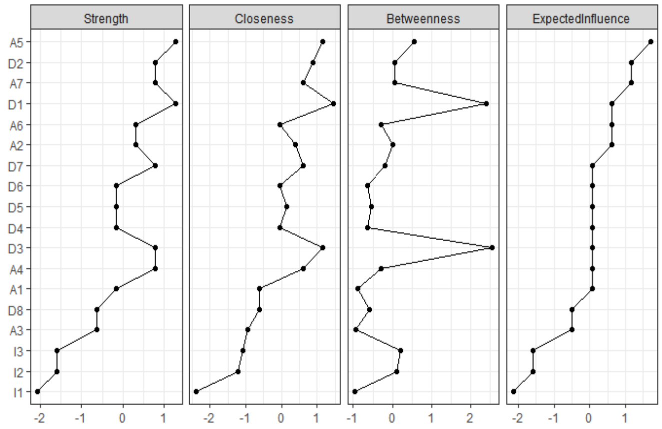

Fig. S16. Bridge centrality indices for psychopathological networks at (a) initial peak , (b) aftermath of initial peak and (c) late COVID-19 phase among individuals in “normal stable” trajectory class . The nodes are ranked in descending order for bridge centrality indices.

(a) Initial peak

Bridge Strength | Bridge Betweenness | Bridge Closeness | Bridge Expected Influence

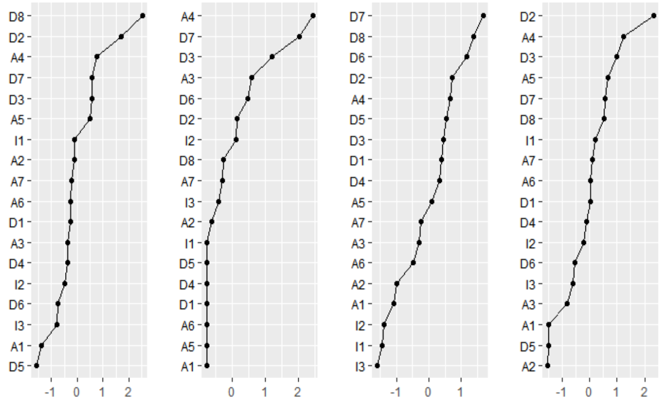

(b) Aftermath of initial peak

Bridge Strength | Bridge Betweenness | Bridge Closeness | Bridge Expected Influence

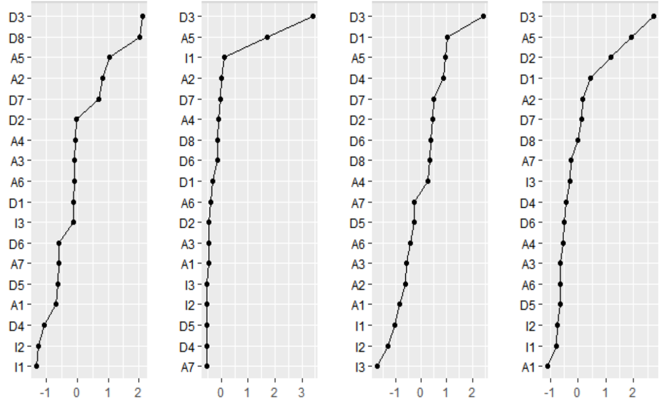

(c) Late COVID-19 phase

Bridge Strength | Bridge Betweenness | Bridge Closeness | Bridge Expected Influence

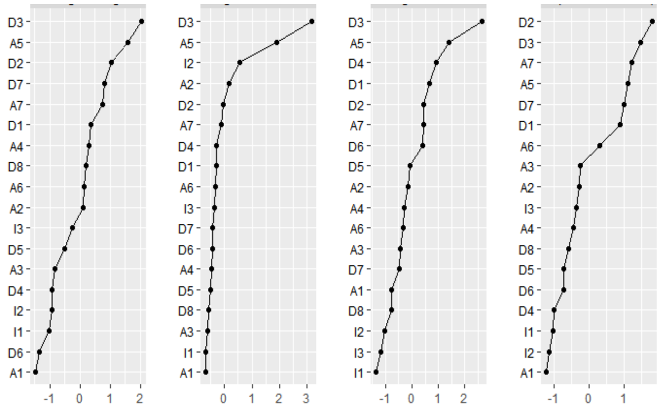

Supplement: Supplementary file 1 [file epssup.zip › S2045796022000518sup002.pdf]
